# Differential localization of dengue virus protease affects cell homeostasis and triggers to thrombocytopenia

**DOI:** 10.1016/j.isci.2023.107024

**Published:** 2023-06-05

**Authors:** Lekha Gandhi, Deepti Maisnam, Deepika Rathore, Preeti Chauhan, Anvesh Bonagiri, Musturi Venkataramana

**Affiliations:** 1 Department of Biotechnology and Bioinformatics, School of Life Sciences, University of Hyderabad, Hyderabad, Telangana, India

**Keywords:** Molecular biology, Virology, Cell biology

## Abstract

Thrombocytopenia is one of the symptoms of many virus infections which is the “hallmark” in the case of dengue virus. In this study, we show the differential localization of existing two forms of dengue virus protease, i.e., NS2BNS3 to the nucleus and NS3 to the nucleus and mitochondria. We also report a nuclear transcription factor, erythroid differentiation regulatory factor 1 (EDRF1), as the substrate for this protease. EDRF1 regulates the expression and activity of GATA1, which in turn controls spectrin synthesis. Both GATA1 and spectrins are required for platelet formation. On the other hand, we found that the mitochondrial activities will be damaged by NS3 localization which cleaves GrpEL1, a co-chaperone of mitochondrial Hsp70. Levels of both EDRF1 and GrpEL1 were found to deteriorate in dengue virus-infected clinical samples. Hence, we conclude that NS2BNS3-mediated EDRF1 cleavage and the NS3-led mitochondrial dysfunction account for thrombocytopenia.

## Highlights

• Dengue virus protease exists in two forms i.e. non-structural protein-3 (NS3) and NS2BNS3
• NS3 localizes to both mitochondria and nucleus whereas NS2BNS3 localizes to nucleus only
• NS3 cleaves GrpEL1 of mitochondrial matrix, and NS2BNS3 cleaves EDRF1 of the nucleus
• EDRF1 cleavage by NS2BNS3 and mitochondrial dysfunction by NS3 account for thrombocytopenia

## Graphical abstract

**Figure:**
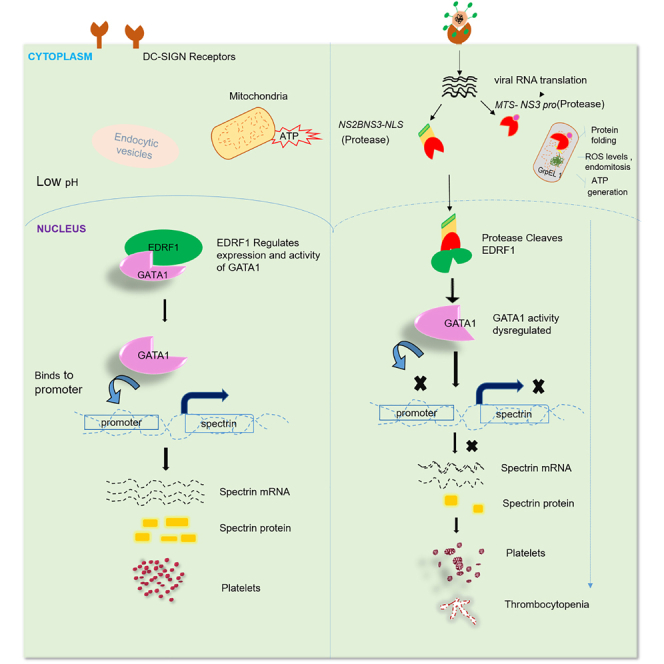

## Introduction

Nearly 130 countries are endemic for dengue virus infections, primarily affecting 2.5 billion inhabitants in the tropical and subtropical regions as well as 120 million travelers to these regions every year. The World Health Organization (WHO) reported an 8-fold increase in dengue cases in the past two decades with approximately 5.2 million cases in the year 2019 alone.^1^^,^^2^ Most of the clinical literature reviews suggest that dengue infection shows asymptomatic or mild symptoms of flu like in dengue fever (DF), severe symptoms like dengue hemorrhagic fever (DHF), and dengue shock syndrome (DSS). Dengue fever manifests symptoms of headache, rashes, muscle soreness, joint pain, retro-orbital pain, and abdominal cramps. DHF is characterized by red or purple blisters on the skin, epistaxis, and gingival bleeding. Thrombocytopenia (low platelet count) and leukopenia were observed mainly during dengue fever with hemorrhagic signs.^3^^,^^4^ In 2009, WHO guidelines defined thrombocytopenia as a major clinical symptom, with a rapid decline in platelet count (i.e., <1, 50, 000 per microliters of blood).^1^^,^^4^

Dengue virus (DENV) is a flavivirus of family *flaviviridae*. There are four distinct serotypes (DENV1-4) that have emerged from sylvatic strains in the forests of Southeast Asia. DENV is an enveloped, single-stranded positive-sense RNA virus. The RNA genome consists of approximately 10,700 nucleotides and codes nearly 3,411 amino acids (a.a) long precursor polyprotein yielding three structural proteins (Capsid (C), precursor Membrane (preM), and Envelope (E) and seven non-structural (NS) proteins (NS1, NS2A, NS2B, NS3, NS4A, NS4B, and NS5).^5^^,^^6^ Among NS proteins that play significant roles during viral replication, NS3 alone or along with NS2B possesses a crucial role and is the prime drug target for developing the anti-virals. NS2B is a 14–15 kDa amphipathic membrane protein and acts as a cofactor for NS3 protein to form an active viral protease complex.^7^^,^^8^ NS2B contains two transmembrane hydrophobic domains and a hydrophilic central domain. The hydrophilic region of 40 amino acid residues linked with G4-S-G4 linker to N-terminal of NS3 protein forming the NS2BNS3pro active protease.^8^^,^^9^ NS3 is a 70 kDa, a multifunctional serine protease forming the catalytic triad with Histidine (H-51), Aspartate (D-75), and Serine (S-135). Its helicase and nucleoside triphosphatase (NTPase) activities are required for unwinding the double-stranded form of RNA.^10^ The N-terminal region forms the protease domain (1–185 residues). NS3 helicase (185–618 residues) belongs to the helical superfamily 2 domain. NS2BNS3pro complex processes polyprotein into mature functional proteins which are required for virus assembly and replication. The C-terminal of NS3 forms a subdomain with essential motifs, subdomain 1 and 2: recombinase A (RecA)like fold and structural motifs required for ATP hydrolysis and RNA binding activities. The other subdomain 3 is involved in forming a single-stranded DNA (ssDNA) binding tunnel during the formation of replication complex.^6^^,^^8^ Cleavage site for DENV protease possesses the dibasic amino acids (Lys-Arg, Arg-Arg, Arg-Lys, Gln-Arg) at P1/P2 followed by a small residue (Gly/Ala/Ser) at P1’.^10^^,^^11^^,^^12^ Reports suggest that the substrate cleavage site for dengue protease is Arg/Lys-Ser-Arg-Ile/Val-Leu at P1-P4’.^13^ Along with cleaving the functional polypeptides, DENV protease is known to cleave host cellular proteins FAM134B (endoplasmic receptor), Ikα/β (cellular factors), Nucleoporins (Nups), mediator of IRF3 activation (MITA), mitofusins (MFN1 and 2), thereby enhancing the viral replication and affecting host metabolism.^14^^,^^15^^,^^16^^,^^17^^,^^18^ During natural infections, dengue virus primarily infects bone marrow cells, myeloid lineages, macrophages, dendritic cells, B and T cells, neuronal cells, and hepatocytes.^19^^,^^20^ The NS protein-3 (NS3) was found to accumulate in phagocytes of spleen and lymph node, liver hepatocytes, and myeloid cells in bone marrow.^21^ Among these cells, bone marrow megakaryocytes (MKs) are highly permissible to infection. Previous reports showed that the dengue virus infection in megakaryocytes with a reduction in cell number with depleting mature megakaryocytes suggests the role of dengue virus in bone marrow homeostasis. Further, it has been reported that the dengue virus abolishes the expression of master transcription factors GATA binding factors 1 and 2 (GATA1, GATA2) and nuclear factor erythroid 2 (NF-E2) which are involved in megakaryocytic developmental processes.^22^^,^^23^ Although the reports of dengue virus infection *in vitro*, *ex vivo*, and *in vivo* models have been studied, the mechanism behind the reduction in platelet cell number causing thrombocytopenia has not yet been clearly understood.^20^^,^^24^^,^^25^

Virus-mitochondria interaction is being revealed in several reports suggesting mitochondrial dysfunction.^26^^,^^27^ Reports also indicate that the virus-coded proteases cleave the mitochondrial proteins. In the case of dengue virus also NS proteins like NS4B and NS2BNS3 target the mitochondrial membranes.^18^ NS3 is reported to target the mitochondrial matrix and cleaves the GrpE Like 1 (GrpEL1), a co-chaperone of mitochondrial heat shock protein (mtHSP70).^28^ The cleavage sites in the dengue virus polyprotein and the nature of the protease activity appear to yield two forms of the protease, i.e., NS2BNS3 and NS3 alone.^12^ In addition to the mitochondrial targeting by the dengue virus protease, it was also detected in the host nucleus.^16^^,^^29^ But whether it is NS2BNS3 or NS3 or both is not known. Hence this study gave an attempt to reveal the localization of NS2BNS3 and NS3, identification of substrates, and consequences with reference to cell homeostasis.

Thrombocytopenia, i.e., reduced platelets is a health disorder that occurs during different ill health conditions including many viral infections which led to the death of millions of people across the globe. Dengue virus infections are known from ancient times which cause “thrombocytopenia” as the primary symptom. Platelet activation, their clearance, and plasma leakage are proposed as the reasons for the platelet reduction in dengue virus patients.^30^^,^^31^^,^^32^^,^^33^^,^^34^ But, to the best of our knowledge, there is no clear study explaining the mechanism involving the virus components in thrombocytopenia. The studies on dengue virus protease (including our report published in the Journal of Virology, 2020^28^) puzzle the scientific community regarding its role in disease pathogenesis. The findings of our present study suggest the localization of dengue virus protease in two subcellular organelles of a cell, i.e., nucleus and mitochondria. There is no such instant indicating the dual localization of a single protein coded by a virus genome. A crucial nuclear transcription factor called EDRF1 (erythroid differentiation regulatory factor 1) is found as the another substrate for this protease. *Ex vivo* and *in vivo* (dengue virus-infected clinical samples) analyses clearly supported the above observation. EDRF1, in turn, regulates another two factors (GATA1 and Spectrins) of the cells (play a key role in proplatelet and platelet formation) which are also downregulated in protease-transfected or virus-infected conditions. Dengue virus-infected K562 cells (megakaryocyte derived) show reduced cell number as the number of days progressed. Mitochondrial dysfunction of the protease-transfected cells was observed which also accounts for the reduction in cell number. Therefore, we felt that this study gave the molecular-level explanation for thrombocytopenia and facilitates focused therapeutic development.

## Results

### Identification of nuclear localization signals (NLSs) and development of recombinant constructs

**Figure 1:**
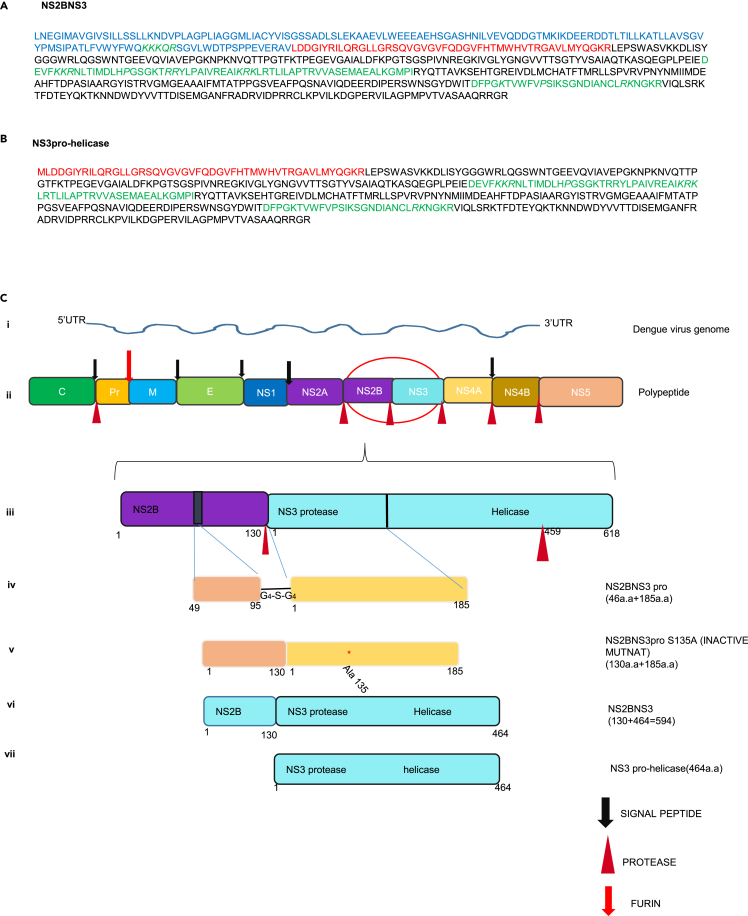
Amino acid sequence of protease and genome organization (A and B) NS2BNS3 (NS2B-130+NS3-464 = 594 a.a) (B) NS3pro-helicase (464 a.a). Amino acid sequence showing the nuclear localization sequence (NLS) in green and mitochondrial targeting sequence (MTS) in red. Full-length NS2B sequence in (blue) and NS3 sequence in (black). (C) (i) Dengue virus genome (RNA) (ii) Dengue virus mature polypeptide showing structural proteins (capsid, pre-membrane, and envelope) and non-structural proteins (NS1 – NS5). (iii) Dengue virus full-length protease with NS2B and NS3 fragments. (iv) NS2B (46 a.a) Glycine linked (G4-S-G4) NS3 protease (185 a.a). (v) Mutant form of NS2BNS3pro (S135A) (NS2B −130 a.a and NS3pro-185 a.a). (vi) NS2BNS3 (130 + 464 a.a) (vii) NS3 pro-helicase (464 a.a).

Using *in silico* method, NLS sequences were identified in both NS2BNS3 (594 a.a) and NS3pro-helicase (464 a.a). In NS2B (130 a.a), three basic amino acid residues ^1471^KKKQR^1475^ at position 1471–1475 were identified which are similar to classical monopartite NLS stretch that consists of basic amino acid residues with motif K (K/R) X (K/R). In NS3 sequence, two stretches of bipartite signal (2–3 positively charged amino acid followed by 9–12 linker sequence containing proline residue) were identified at positions (a.a 1656–1716 and 1839–1856) with a score of 4–4.1 (Figures 1A and 1B). The mitochondrial targeting sequence (MTS) was identified in NS3 during our earlier study.^28^ Based on these analyses the recombinant constructs NS2BNS3pro (46 + 185 a.a), NS2BNS3pro (S135A) mutant (130 + 185 a.a), NS2BNS3 (594 a.a), and NS3pro-helicase (464 a.a) were generated (Figure 1C) as detailed in methods.

### Protease localization studies

**Figure 2:**
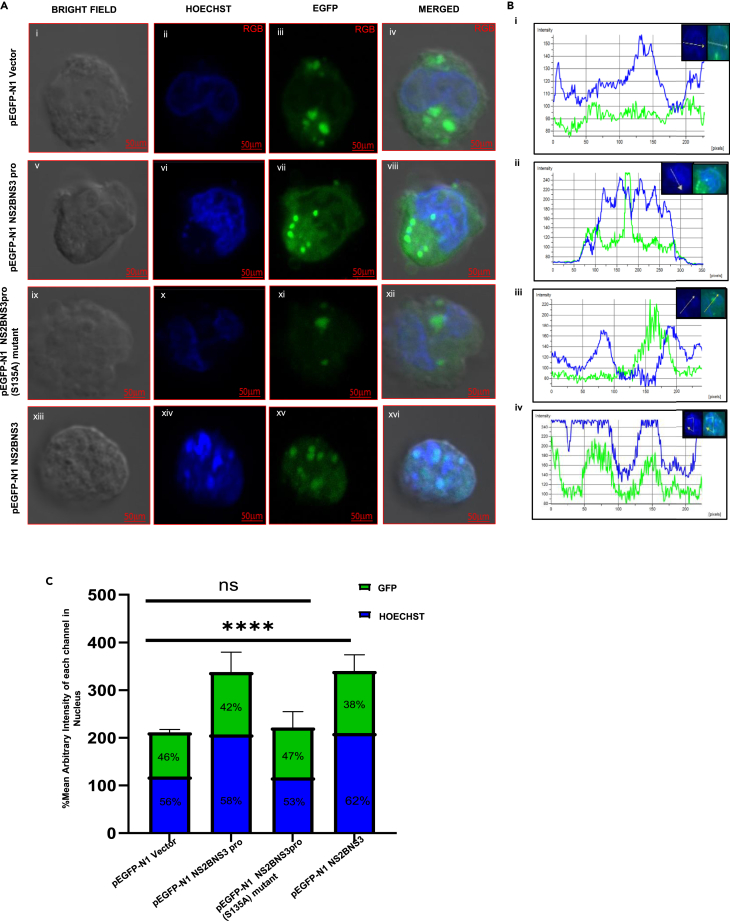
Localization of expressed dengue virus protease in K562 cells (A) pEGFP-N1 vector (i-iv), pEGFP-N1 NS2BNS3pro (v-viii), pEGFP-N1 NS2BNS3pro (S135A) mutant (ix-xii), and pEGFP-N1 NS2BNS3 (xiii-xvi) were transfected transiently and observed after 28 h post transfection. The images represent the bright field (Gray), Hoechst33342 (nuclear staining blue), GFP (green) and merged at 100X (scale bar = 50μm) magnification using Carl Zeiss confocal microscopy. See also Figure S1. (B) i. Fluorescence intensity profiles using NIS Elements AR software of pEGFP-N1 vector showing GFP expression (green) in cytoplasm. ii-iv. pEGFP-N1 NS2BNS3pro, pEGFP-N1 NS2BNS3pro (S135A) mutant, and pEGFP-N1 NS2BNS3 show the expression in nucleus with different intensities. (C) The bar graphs represent the percentage arbitrary mean intensity of each channel (Green and Blue) in nucleus of transfected cells presented as mean values (±) SD plotted in graph pad prism 9. The percentage was calculated using (mean intensity of each channel/total intensity in nucleus X100). *p* values indicated p < 0.0001, ∗∗∗∗ = significant, ns = non-significant.

We performed transfections in K562 cells and analyzed at 20× magnification for the transfection efficiency, and the data suggested that 50–60% of the cells are positively transfected (Figure S1). Further, the data indicated that the expressed proteins (GFP-tagged) of all three constructs (NS2BNS3pro, NS2BNS3pro (S135A) mutant, and NS2BNS3) localized to the nucleus compared to vector alone as the GFP was found to merge with the Hoechst stain (nucleus) (Figure 2A [viii, xi and xvi]). In the enhanced green fluorescent protein plasmid N-terminal (pEGFP-N1) vector, the intensity of the green peak is low and does not merge with blue peak, but in case of pEGFP-N1 NS2BNS3pro, pEGFP-N1 NS2BNS3pro (S135A) mutant and pEGFP-N1 NS2BNS3 show merged green peaks with blue peaks of varying intensities indicating the GFP expression in nucleus (Figure 2B [i-iv]). The bar graphs further support the above observation with the high GFP/blue expression ratios which represent the percentage mean arbitrary intensity in the nucleus (Figure 2C).

**Figure 3:**
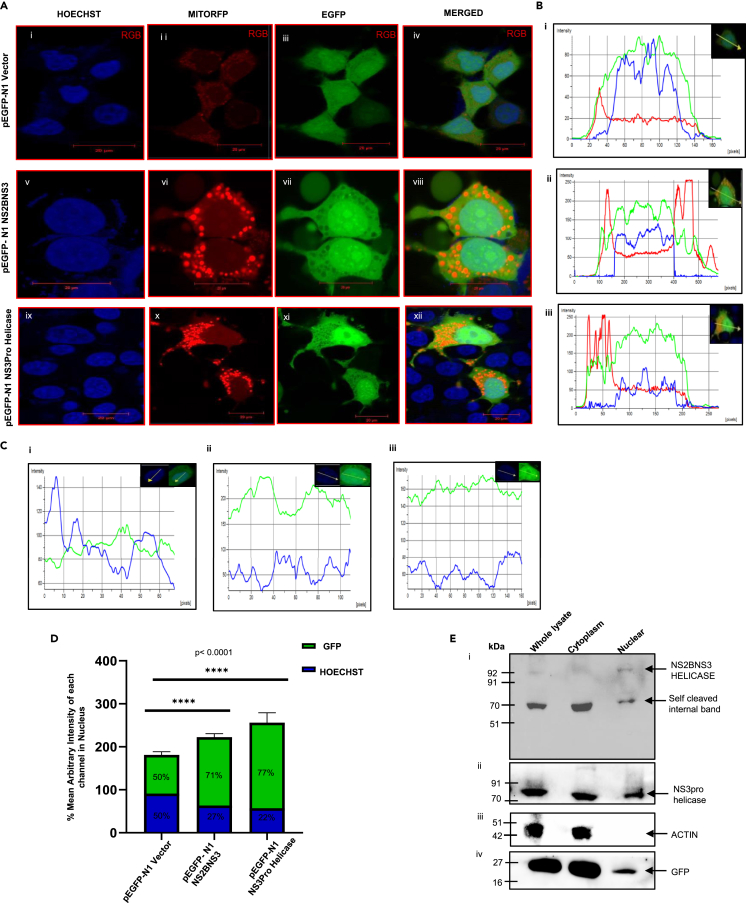
Localization of NS2BNS3 and NS3pro-helicase (A) Confocal images at 60X (scale bar = 20μm) showed the localization of pEGFP-N1 vector (i-iv); pEGFP-N1 NS2BNS3 (v-viii); and pEGFP-N1 NS3pro-helicase (ix-xii), with both localization markers, i.e., MitoRFP and Hoechst. (B) i. Fluorescence intensity profiles were generated using NIS Elements AR software of pEGFP-N1 vector, ii. pEGFP-N1 NS2BNS3, iii. pEGFP-N1 NS3pro-helicase. (C) i-iii Represent the intensity profiles of GFP (green) in nucleus (blue). (D) The bar graph representing the arbitrary mean intensity of each channel (green and blue) in nucleus of transfected cells as calculated in Figure 2C. (E) pEGFP-N1 NS2BNS3 and NS3pro-helicase subcellular fractions of transfected HEK cell lysates probed with NS2BNS3pro antibody.

The NS2BNS3 also possesses the MTS along with NLS; hence we intended to analyze the possibility of its localization to the mitochondria also. In this direction, the NS2BNS3 was transfected along with mitochondria red fluorescent protein (MitoRFP), stained with Hoechst stain and analyzed for the localizations. The data suggested GFP and the MitoRFP expression are not merged suggesting no localization of NS2BNS3 to the mitochondria (Figure 3A [viii]). This observation is further supported by the graphical analysis showing that the red peaks are completely separated from the green peaks (Figure 3B ii). It was further confirmed that NS2BNS3 localized in nucleus as indicated with a high-intensity peak of GFP (green peak) in the nuclear region (Figure 3C [ii]). But NS3 alone was reported to be localized to the mitochondrial matrix which shows both the MTS and NLS. In order to properly understand the localization of NS3 also, we carried out the transfections along with MitoRFP, stained with Hoechst stain, and analyzed the localizations in mitochondria and nucleus. The results show that NS3pro-helicase enters both mitochondria and nucleus as indicated by the intensity profiles where the GFP expression (green) is merging with red (mitochondria) and high GFP intensity in blue peak region (nucleus) (Figures 3A [xii]; 2B [iii]; 3C [iii]). The bar graphs which represent the percentage mean arbitrary intensity of each channel in the nucleus indicated the GFP expression in nucleus supporting the above observations (Figure 3D).

With the cell lysates of the above experiments, we have carried out the western blotting analysis (anti-NS2BNS3pro antibody) to verify the presence of protease forms that localize to nucleus or mitochondria or both. The immunoblotting results were found to be consistent with the above localization experiments suggesting that the NS3pro-helicase (75 kDa) localized to both cytoplasm (mitochondria) and nucleus (Figure 3E ii), whereas NS2BNS3 (92 kDa) localized only to the nucleus (Figure 3E i). We also observed a 70 kDa band in the nucleus (Figure 3E i), possibly due to an internal self-cleavage site in NS2BNS3. Absence of actin (Figure 3E iii) and less GFP (Figure 3E iv) in nuclear fraction support the fractionations.

### Identification of protease-interacting proteins

**Figure 4:**
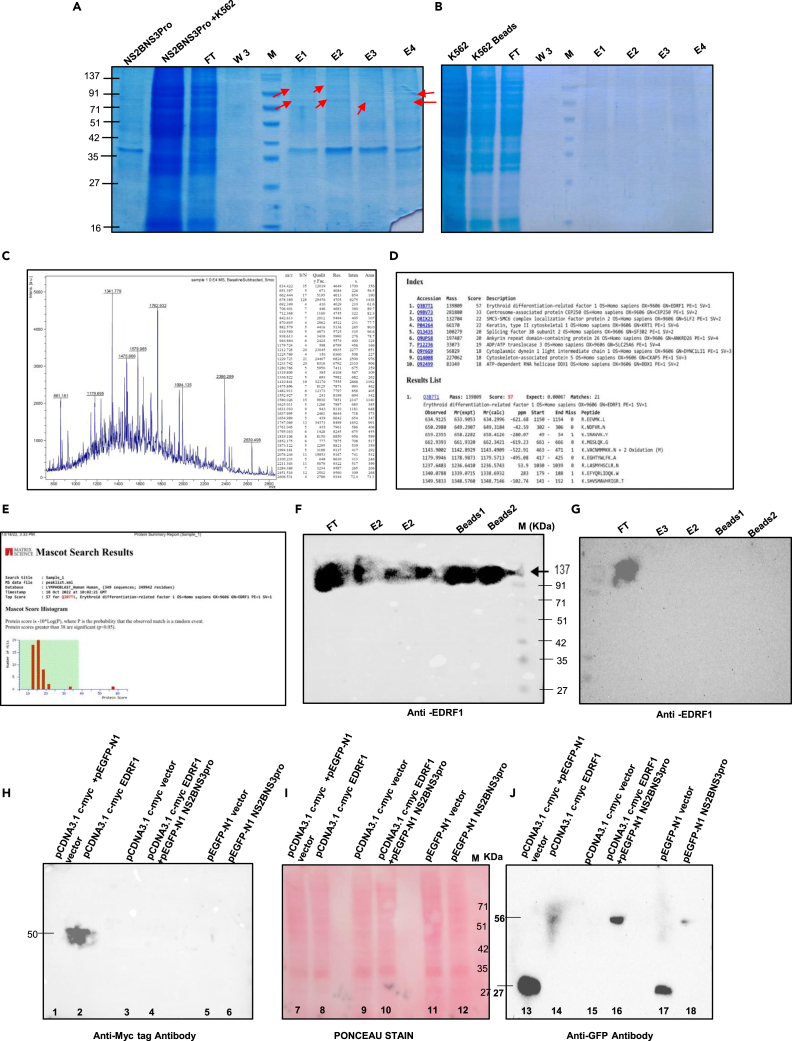
Identification of EDRF1 as another substrate of dengue virus protease (A and B) *In vitro* pull-down assay from K562 cell lysate with pRSET-A NS2BNS3pro purified protein and (B) negative control. The arrows indicate the bands used for identification. See also Figures S2A and S2B. (C and D) Identification data of the protein bands obtained from above *in vitro* pull-down assay. (E–G) The graph represents the Mascot search result showing the identified protein as EDRF1 with the top score 57. Western blotting analysis of the fractions of pull-down assay by using anti-EDRF1 antibody, experimental (F), and negative control (G). (H) Western blot analysis after 48 h of co-transfection. HEK cells were transfected with single or co-transfected with recombinant vectors as indicated on the blots and probed with anti-myc tag antibodies. (I) Ponceau S staining of the above membrane Lanes: 7–12 and M (protein marker). (J) The above membrane was stripped and probed with anti-GFP antibodies.

To identify the host factors interacting with NS2BNS3 protease, we performed *in vitro* nickel-nitrilotriacetic acid (Ni-NTA) pull-down assay with purified NS2BNS3pro incubating with K562 whole cell lysate (Figures 4A, 4B, S2A and S2B). Protein bands that appeared in elutes (E1-E4) were identified by matrix-assisted laser desorption ionization-time of flight (MALDI-TOFF) mass spectrometry. The identified bands from the above elutes were found to be EDRF1 (Figures 4C and 4D). Also, the Mascot search result supported the above finding that the identified protein EDRF1 has a score of 57 which is the highest among the list (Figure 4E). Western blot analysis using anti-EDRF1 antibody showed the presence of EDRF1 in elutes and beads thus confirming EDRF1 as a protease-interacting host protein (Figures 4F and 4G).

### Identification of protease cleavage sites in EDRF1 using *in silico* methods

ProP-1.0 server identified a total of five cleavage sites (dibasic amino acid residues, i.e., Arg/Lys in P1/P2 position) at different amino acid sequence positions of EDRF1 (50,100,152,926,987) (Figure S2C). In order to know which of the above cleavage site is located near the catalytic triad of protease, we performed *in silico* docking method for analyzing the interactions of protease and EDRF1 by superimposed models (Figure S3A). It was observed that the cleavage site at a.a 985–988 of EDRF1 (cyan) interacted with the catalytic triad of protease (H-51, D-75, S-135) (green) (Figure S3B). Thus, we have developed a recombinant plasmid cloning DNA 3.1 (pcDNA3.1) c-myc vector containing the c-terminal end from amino acid 798 to 1238, approximately 50 kDa of EDRF1 (encompassing a.a 985–988 cleavage site) as described in STAR Methods.

### EDRF1 cleavage in co-expressed conditions

The cell extracts co-expressed with both EDRF1 and protease were resolved on 10% SDS PAGE and transferred onto the hydrophilic polyvinylidene fluoride (PVDF) membrane. Western blot analysis was done to detect the levels of EDRF1 using anti-Myc tag antibody. The blot showed no band in co-transfected vectors as the Myc tag is only 1.2 kDa (very small to detect) (Figure 4H lane 1). In pcDNA3.1 c-myc containing EDRF1 as an insert, the EDRF1 band was detected as intact showing the presence of overexpressed EDRF1 alone (Figure 4H lane 2). As expected, no expression was observed in pcDNA3.1-myc vector alone (Figure 4H lane 3). Importantly, EDRF1 completely disappeared in presence of protease in co-transfected pCDNA3.1 c-myc EDRF1 and pEGFP-N1 NS2BNS3pro, suggesting EDRF1 as a substrate of protease (Figure 4H lane 4). pEGFP-N1 vector alone and pEGFP-N1 NS2BNS3pro lysates were loaded as controls (Figure 4H lanes 5 and 6).

To confirm the expression of NS2BNS3pro in the above experiment, we have checked the expression of protease using anti-GFP antibody (pEGFPN1-NS2BNS3pro) after stripping the same membrane. It was observed that, in co-transfected vectors and pEGFP-N1 vector alone, GFP was expressed (Figure 4J lanes 13 and 17). No band was observed in pCDNA3.1 c-myc EDRF1 and pCDNA3.1 c-myc vector alone using anti-GFP antibody (Figure 4J lanes 14 and 15). In co-transfected pcDNA3.1 c-myc EDRF1 and pEGFP-N1 NS2BNS3pro cell lysates, the protease was detected thus confirming the expression of NS2BNS3pro in the co-transfected conditions (Figure 4J lane 16). pEGFP-N1 NS2BNS3pro was also found to be expressed alone as a control (Figure 4J lane 18).

### Analysis of levels of EDRF1, GATA1, and spectrins in protease-transfected and virus-infected cell lysates

**Figure 5:**
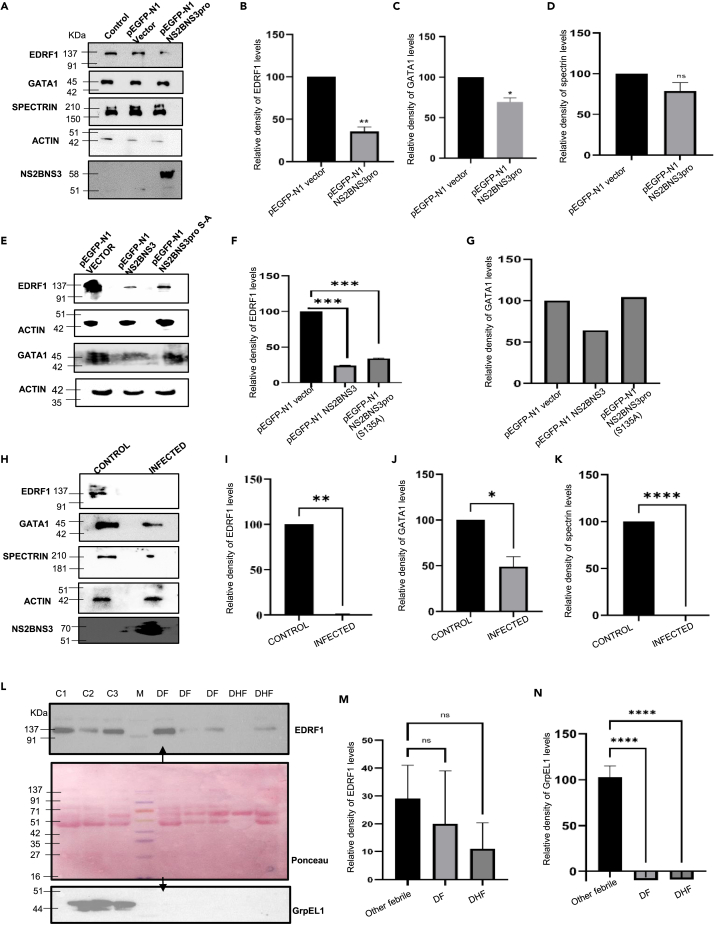
Analysis of levels of EDRF1, GATA1, spectrin, and GrpEL1 in *ex vivo* and *in vivo* (clinical samples). Western blot analysis of K562 transfected cell lysate (A) pEGFP-N1 vector and pEGFP-N1 NS2BNS3pro for 28 h of transient transfections with indicated antibodies on the left. (B–D) Bar diagram represents the means of 3 independent sets of experiments for proteins indicated. (E) Western blot analysis of K562 cell lysate transiently transfected with pEGFP-N1 NS2BNS3 and pEGFP-N1 NS2BNS3pro (S135A) mutant analyzed after 28 h of transfection with indicated antibodies. (F–G) Bar diagram represents the means of three sets of independent experiments for the indicated proteins. (H) Western blot analysis of DENV-infected and uninfected in K562 cell lysates after 7 days with indicated antibodies. (I–K) Bar diagram of respective proteins of 3 sets of independent experiments. (L) Albumin out other febrile (C1, C2, C3) and dengue-infected serum samples (DF and DHF). The samples were separated on 10%SDS PAGE, and western blotting was done with anti-EDRF1 (top) and anti-GrpEL1 (bottom) antibodies and Ponceau stained membrane (Middle). (M and N) Bar diagrams represent the relative densities of EDRF1 and GrpEL1 proteins in clinical samples. See also Figure S4 and Table S1.

The GATA1 expression and activity were reported to be regulated by EDRF1.^35^^,^^36^^,^^37^ GATA1 in turn controls the synthesis of spectrin proteins which are required for pro-platelet and platelet formation.^38^ EDRF1, a 138 kDa protein, is reported to be expressed in megakaryocytic (K562) and erythroid cell lineages.^35^^,^^36^ Hence, we intended to analyze the levels of EDRF1, GATA1, and spectrins in protease-transfected and virus-infected K562 cells. For this purpose, total cell lysates of transfected/infected were resolved on 10% SDS PAGE followed by western blot analysis with anti-EDRF1 antibody. It was observed that EDRF1 levels were deteriorated significantly in presence of pEGFP N1 NS2BNS3pro as compared to the vector, concluding that EDRF1 was being cleaved by dengue virus protease (Figures 5A–5D). The same cell lysates were used to check the endogenous levels of GATA1 and spectrins. It was observed that, compared to pEGFP N1 vector alone, GATA1 and spectrin levels were found to be reduced (Figures 5A–5D). To confirm the above data, we have transfected pEGFP N1 NS2BNS3 (594 a.a) which represents one of the forms of naturally existing dengue virus protease. A mutant of NS2BNS3pro (NS2BNS3pro S135A) carrying the mutation in the catalytic triad was also included. The data suggest that the levels of EDRF1 and GATA1 were drastically reduced in presence of pEGFP N1 NS2BNS3, compared to the mutant and the vector (Figures 5E–5G).

To further confirm the above result, we extended our study using the K562 virus-infected cell lysates (7 days post infection) as mentioned in STAR Methods. Supporting the transfection analyses, the levels of EDRF1, GATA1, and spectrin proteins were compromised significantly, clearly depicting the role of protease in EDRF1 cleavage (Figures 5H–5K). It was observed that the reduction of EDRF1 levels is clear in virus-infected cell lysates compared to the above transfection studies.

### Analysis of EDRF1 and GrpEL1 levels in clinical samples

Total forty-four albumin out clinical samples with different grades of infections, i.e., DF, DHF, and DSS were included for the analysis (Table S1). Other febrile-infected samples were used as controls. SDS PAGE followed by western blotting was done to analyze the levels of EDRF1. It was observed that EDRF1 levels were reduced in DF, DHF, and DSS samples, but a significant difference was observed in DHF and DSS samples (Figures 5L [upper panel] and 5M; Figure S4). GrpEL1 was also included in this study as this protein was identified as a substrate of NS3 in our earlier studies.^28^ The GrpEL1 levels were also found to be reduced (consistent with our earlier report) in the samples in which the EDRF1 levels were reduced, i.e., in case of DF and DHF (Figure 5L [lower panel] and N).

### Effect of protease on mitochondrial functions

NS3 was shown to target the mitochondrial matrix and cleave GrpEL1, a co-chaperone of mitochondrial Hsp-70 (mtHsp-70), in our previous study.^28^ In the present study also it was observed that NS3 localizes to mitochondria as well as into the nucleus (Figure 3A [ix-xii]). In this direction, to analyze the functional activities of mitochondria we have used HepG2 cell lines (hepatocytes) that have moderate energetic demand and are frequently used for several mitochondrial studies.^39^ They are also reportedly permissible for dengue virus infections.^40^ Thus, in this study we have performed cell mitostress assays for pEGFP-N1 vector and pEGFP N1 NS3pro-helicase-transfected HepG2 cells, and six parameters were measured as described below.

### Basal respiration

**Figure 6:**
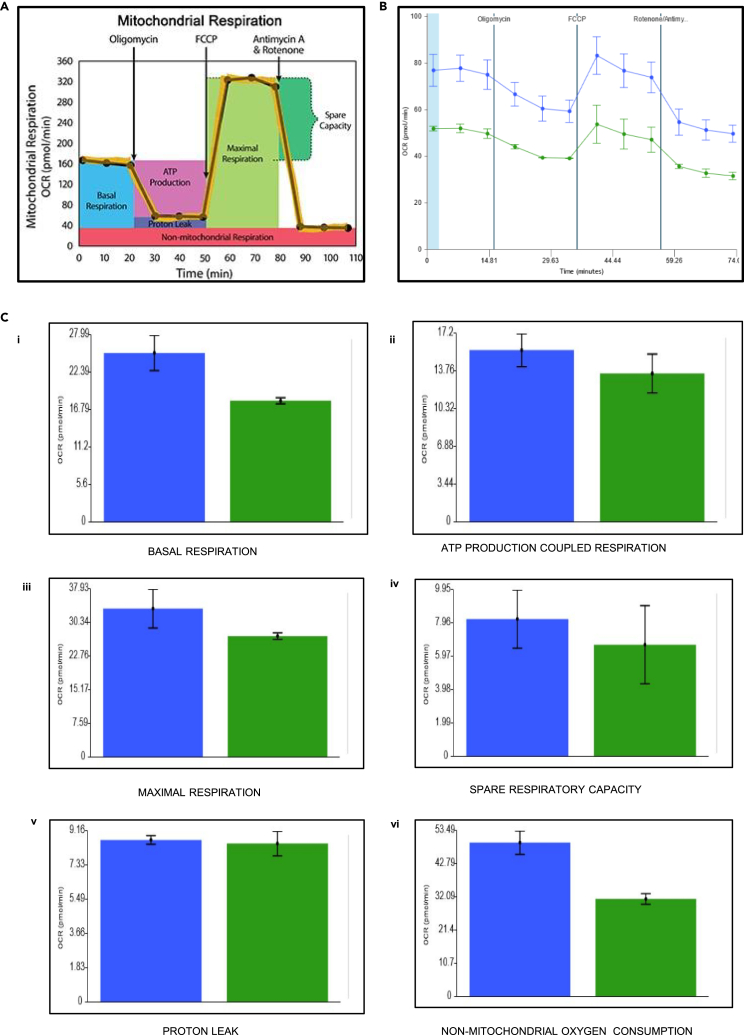
Mitochondrial function analysis in protease-transfected cells (A) Represents the reference model for the analysis of mitochondrial bioenergetics in mitostress assay. (B) Agilent Seahorse XFp cell mitostress assay showing bioenergetics of mitochondria in cells transfected with pEGFP-N1 vector and pEGFP-N1 NS3pro-helicase with respect to oxygen consumption rate vs. time. (C) The graphs represent the oxygen consumption rate vs. time during injection of the three drugs (Oligomycin, FCCP, and Rotenone/Antimycin). (i) Basal respiration (ii) ATP production-coupled respiration (iii) Maximal respiration (iv) Spare respiratory capacity (v) Proton leak (vi) Non-mitochondrial oxygen consumption. The parameters measured are shown in representative bar graphs with error bars of at least two experiments (n = 3, i.e., triplicates in each experiment).

Oxygen consumption rate (OCR) is a measure of the total cellular respiration in cells that meet the cellular ATP demand of the cell under baseline conditions. Basal levels of OCR in NS3pro-helicase-transfected cells were found to be decreased compared to vector-transfected cells which suggested low mitochondrial respiration in transfected cells (Figure 6C i).

### ATP production-coupled respiration

On the addition of oligomycin, F0/F1 ATPase (complex V) shuts off, which relates to the activity of mitochondria generating the ATP linked to basal respiration. It was observed that in presence of oligomycin, the ATP production was compromised in NS3pro-helicase-transfected cells when compared to vector (Figure 6C ii). A decrease in ATP production-coupled respiration results in low ATP demand indicating damage to the electron transport chain (ETC).

### Maximal respiration

On the addition of Carbonyl cyanide p-(trifluoromethoxy) phenylhydrazone (FCCP) (uncoupler), the movement of protons (H+ ions) will be allowed. This phenomenon leads to sudden increase in maximal respiration indicating high substrate availability and good integrity of the ETC as observed in vector-transfected cells. However, there was no increment in maximal respiration in NS3-pro helicase-transfected HepG2 cells (Figure 6C iii) indicating the defective mitochondrial ETC.

### Spare respiratory capacity

It is a measure of the difference between maximal respiration and basal respiration and represents the cell fitness and the cell response due to high energy demand. In NS3pro-helicase-transfected cells, maximal respiration was found to be low compared to vector; thus there was low spare respiratory capacity compared to the vector. Low respiratory capacity indicates low fitness of the cell (Figure 6Civ).

### Proton leak

Basal respiration that is not linked to ATP production indicates the proton leak. There was no significant change in proton leak due to low energy demand or low substrate availability in transfected cells (Figure 6C v).

### Non-mitochondrial oxygen consumption

Addition of antimycin and rotenone inhibits complex III leading to low cellular respiration. Non-mitochondrial oxygen consumption is the measure of the cells recovering their energy demand through other cellular processes. In the NS3pro-helicase-transfected cells, there was no increase in non-mitochondrial OCR as compared to the vector indicating the damage to mitochondria (Figure 6C vi).

### Reduction in cell number during dengue virus infection in K562 cells

**Figure 7:**
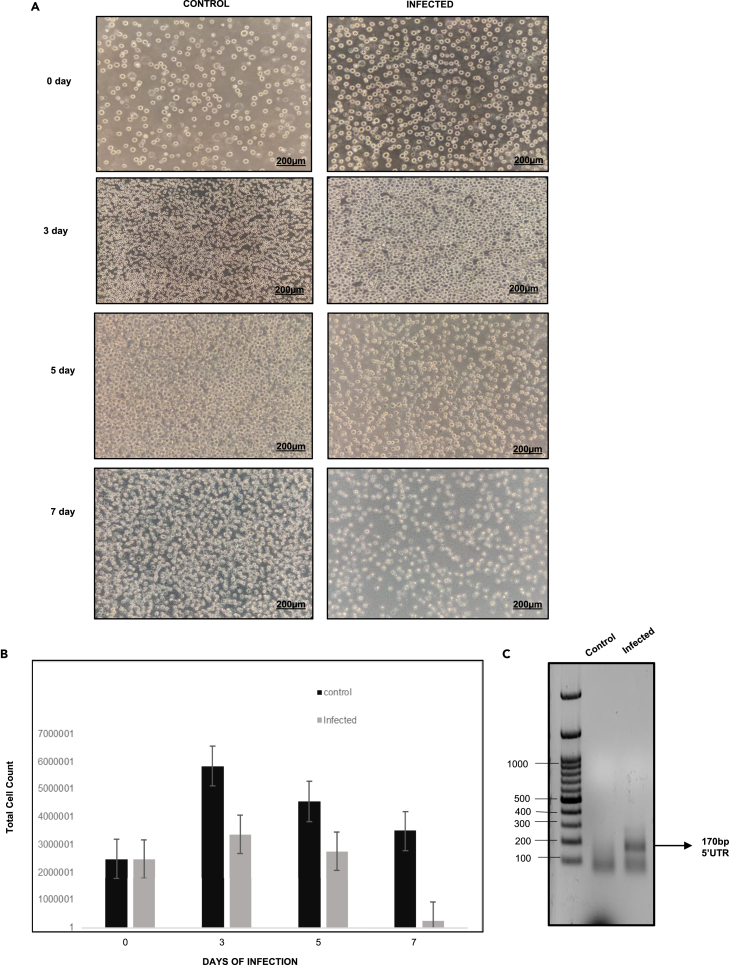
Analysis of cell number during dengue virus infection (A) Morphological analysis at 10X (scale bar = 200μm) of infected and uninfected K562 cells observed at different days post infection from 0 to seventh day. (B) Bar graph showing the total cell count of uninfected and infected K562 cells. The error bars represent the average total cell count of at least two independent experiments. (C) RT-PCR analysis of amplified fragment of 5′UTR (170 bp) during dengue infection on the fifth day.

In order to analyze the effect of virus infection on K562 cells (megakaryocyte-derived cell line), we performed a cell counting method to analyze the cell number during virus infection. For this purpose, K562 cells with dengue-infected or uninfected were analyzed day wise (0, 3, 5, and 7) for morphological changes. Fourth day post infection onwards, visible cell morphological changes (deformed shape, reduced size, and tiny appearance) occurred. Total cell count starts decreasing after day 3. The rounded cell morphology of K562 cells was changed, a visible reduction in cell size on seventh day was observed, and the changes were imaged under an inverted bright-field microscope (magnification at 10X) (Figures 7 A and 7B). The virus infection was confirmed by RT-PCR on the fifth day (Figure 7C).

## Discussion

Dengue virus infections occupied the top of the list of emerging infectious diseases at present, but there is no specific drug or vaccine until now. All four serotypes cause similar disease manifestations individually or in combination with other serotypes. Flaviviruses target many host cellular proteins for efficient viral replication. The proteases of dengue and other flaviviruses (West Nile virus [WNV], Japanese encephalitis virus [JEV], and Zika virus) are known to cleave self-polyprotein and host proteins.^8^^,^^41^ The polyprotein cleavage sites in case of dengue virus and its nature of activity suggest the possibility of existence of two forms of protease, i.e., NS3 alone and along with NS2B, i.e., NS2BNS3.^12^ NS2BNS3 is reported to target the mitochondrial membrane whereas NS3 alone targets the mitochondrial matrix in which the MTS was identified.^18^^,^^28^ The protease was also found in the nucleus without referring to any of the above two forms.^29^ Hence as a first step in this study, we have searched for the NLSs in both the NS2BNS3 and NS3. The data suggested the presence of NLS in both the above two forms (Figures 1 A and 1B). A similar stretch with (KKKRK) was found to be reported in simian virus 40 (SV40) large T-antigen.^42^ Transfection experiments carried out in order to verify the above observation with the developed recombinant constructs indicated the localization of NS2BNS3 into the nucleus and NS3pro-helicase localization into both the mitochondria and the nucleus (Figures 2 and 3). The mitochondrial localization of NS3 is consistent with our earlier observations.^28^ In order to further confirm the above observations, we have carried out the transfection experiments using the mitochondrial (MitoRFP) and nuclear (Hoechst stain) localization markers for both NS2BNS3 and NS3. The results show that, while the NS3pro-helicase localized with the Hoechst stain (nucleus), NS2BNS3 did not colocalize with the MitoRFP (mitochondria) which supports the above observations (Figure 3). It was speculated that the NS2BNS3 could not enter the mitochondria as the MTS present at the N-terminal end of the NS3 is masked by NS2B. Both NS2BNS3 and NS3 localized to the nucleus due to multiple NLSs present in both forms. The western blot analysis carried out to detect the protease in the nuclear or the cytoplasmic (mitochondria) extracts indicates the presence of the NS3 (75 kDa) in cytoplasmic (mitochondria) fraction whereas both NS2BNS3 and NS3 (92 & 75 kDa) were present in the nucleus (Figure 3E). A 70 kDa band observed in the nucleus of NS2BNS3-transfected extracts may be due to the internal self-cleavage site (Figure 3E [i]).^12^ The above observations suggest the localization of both the NS2BNS3 and NS3 into the nucleus.

In order to identify the substrates of NS2BNS3/NS3, if any, from the nucleus, we have carried out the pull-down experiment consisting of total cell extracts with NS2BNS3pro. The co-eluted protein bands were identified by mass spectrometry analysis and were found to be EDRF1 (Figures 4A–4E, S2A, and S2B). Western blotting analysis of the elutes of the above experiment using anti-EDRF1 antibodies confirms the interaction of NS2BNS3 with EDRF1 (Figures 4F and 4G). In continuation, the amino acid sequence of ERDF1 was searched for the cleavage sites, and we found five sites at amino acid sequences 50,100,152,926,987 (Figure S2C). Structural superimposition of EDRF1 and protease suggested that the cleavage site (RK/A) (985–988) is located in the catalytic triad of protease (Figure S3). Hence, we have generated the EDRF1 recombinant construct from the amino acids 798–1238 of EDRF1. Co-transfection experiments carried out with the above EDRF1 and the NS2BNS3pro constructs suggested the complete disappearance of EDRF1 but not in controls (Figure 4H, lane 4). This observation suggested EDRF1 as another substrate of dengue virus protease, which reportedly regulates the expression and activity of GATA1.

GATA1 is involved in differentiation, proliferation, and maturation of megakaryocytes (platelet lineage), and its activity is modulated by the EDRF1 protein. EDRF1 has been reported to play a major role in transcriptional regulation and expression of GATA1 and globin gene expression, a key factor in megakaryopoiesis.^43^ Based on these reports we framed the hypothesis that EDRF1, GATA1, and spectrins might be affected during the dengue virus infection. In order to test the same, the NS2BNS3 transfection experiments carried out in K562 cells suggested the reduced levels of EDRF1, GATA1, and spectrin proteins in the expressed conditions of protease (Figures 5A–5G). Similar analysis carried out using the cell extracts of the dengue virus-infected cells supported the above observations (Figures 5H–5K). Both the above two conditions strongly suggest the cleavage of EDRF1 and as a result the downregulation of GATA1 and spectrin proteins. Further, the EDRF1 and the GrpEL1 levels were analyzed in the dengue virus-infected clinical samples. GrpEL1 was also chosen, as it was shown to be the substrate of NS3 in our earlier studies.^28^ The levels of both the above proteins were significantly reduced in dengue virus-infected samples which are drastic in case of the severe disease (DHF and DSS), indicating the cleavage of ERDF1 (Figures 5L–5N and S4). The effect on the EDRF1 levels is more and more clear from *ex vivo* to *in vivo* (clinical samples) conditions (Figures 5 and S4). Reports indicate that EDRF1 plays a key regulatory role in GATA1 mRNA expression and DNA binding activity.^35^^,^^36^ Importantly, GATA1 is known to be involved in erythrocyte and megakaryocytic precursor development; disruption of any of the above-mentioned two functions of GATA1 leads to thrombocytopenia and anemia.^43^^,^^44^ Several lines of reports indicate that defects in GATA1 activity lead to thrombocytopenia, structural abnormalities in megakaryocytes, and impairment of platelet activation.^22^^,^^43^^,^^44^ GATA1 is also known to involve in spectrin mRNA synthesis, and the spectrin proteins are reportedly needed for pro-platelet and platelet formation.^45^ Several lines of research indicated that alpha and beta spectrins are essential proteins involved in cytoskeleton formation, particularly in erythrocytes, in association with actin.^46^ Interesting function of spectrin proteins, as far as the present study concerned, is their involvement in the pro-platelet and platelet formation. Hence the cleavage of EDRF1 observed in the present study led to decreased levels of GATA1 and subsequently to fewer spectrins and finally to the reduced number of platelets, i.e., thrombocytopenia. The proposed mechanisms of thrombocytopenia in dengue virus infections^30^^,^^31^^,^^32^^,^^33^^,^^34^ and present observations are possibly parts of a cascade of events of a single mechanism. However, further research is required to indicate whether they are interlinked or different and which is predominant, if different. Non-functional platelets without the number change (thrombopathy) are another possible disorder to be considered in dengue virus-infected patients. But, as the current literature including the present study support the reduction in platelet number, the “thrombopathy” may need to be further assessed.

Reports suggest defective mitochondrial quality and dysfunctioning during viral infections including dengue virus.^26^^,^^34^ Reports also indicate that the dengue virus NS2BNS3 targets the mitochondrial membrane proteins and NS3 enters the mitochondrial matrix and cleaves the matrix protein (GrpEL1), a co-chaperone of the mtHSP70.^28^ In all the above cases, the role of the above proteins in mitochondrial activity is not clear. Hence, we have analyzed the activities of mitochondria (basal respiration, ATP production-coupled respiration, maximal respiration, spare respiratory capacity, proton leak, and non-mitochondrial oxygen consumption) in the cells transfected with NS3. These activities were found to be reduced (except proton leak) significantly compared to the controls (Figure 6). This observation supported the hypothesis that the mitochondrial dysfunction/defectiveness is due to the effect of the dengue virus-coded protease. The above findings related to mitochondria were supported by the earlier studies carried out by virus infections.^40^ Mitochondrial dysfunctions are also reported to be leads to impaired platelet formation.^34^ Hence, we proceeded with the analysis of cell numbers under dengue virus infections using the megakaryocyte-derived K562 cells. The results showed a reduction in the number which is more prominent as the number of days progressed (Figure 7). Platelets are the cell types that are required in a huge number in the blood at a given point of time. In order to produce a large number of cells, the platelet lineage cells have to undergo vigorous growth and differentiation which require a high input of ATP. But the mitochondrial damage occurred during dengue virus infections (due to protease as described above), and the cells are incapable of dividing fast to yield enough platelet number because of less ATP generation by mitochondria. Hence, it is concluded that cleavage of the EDRF1, a nuclear transcription factor, by NS2BNS3 of dengue virus, and the NS3-mediated mitochondrial dysfunction contribute to the reduced number of platelet formation (thrombocytopenia) during dengue virus infections.

### Ethics statement

This study was approved by the Institutional Ethics Committee, University of Hyderabad (UH/IEC/2021/1).

### Limitations of our study

The present study carried out is completely around the dengue virus protease and its effect on cell homeostasis. Availability of a specific anti-protease molecule would have shed light more clearly on the observations. But the reported two anti-protease drugs quercetin and Nordihydroguaiaretic acid (NDGA) are highly cell toxic and hence were not used in this study. Hence the availability of a specific anti-protease molecule could be considered as the limitation in the present study.

## STAR★Methods

### Key resources table

**Table:** 

REAGENT or RESOURCE	SOURCE	IDENTIFIER
**Antibodies**		

EDRF1 Rabbit Polyclonal Antibody	Proteintech, USA	Cat# 21883-1-AP, RRID: AB_11042452
MYC tag Mouse Monoclonal Antibody	Proteintech, USA	Cat# 60003-2-Ig, RRID: AB_2734122
Alpha-1 Spectrin Rabbit Polyclonal Antibody	ImmunoTag	Cat# ITT13417 RRID: N/A
GATA-1 Rabbit Polyclonal Antibody	ImmunoTag	Cat# ITT06184 RRID: N/A
GFP (D5.1) Rabbit mAB(monoclonal antibody)	Cell Signaling Technology	Cat# 2956, RRID:AB_1196615
Actin mouse Monoclonal Antibody	Santa Cruz Biotechnology	Cat#sc8432
Rabbit Anti-Mouse-IgG HRP conjugated Antibody	GeNei	Cat# 1140580011730 RRID:N/A
Mouse Anti-Rabbit- IgG HRP Conjugated Antibody	Santa Cruz Biotechnology	Cat# sc-2357, RRID:AB_628497
NS2BNS3pro Rabbit Antibody (raised in housed)	(Gandikota et al.^47^)	N/A

**Bacterial and virus strains**		

*Escherichia coli*. (BL21 competent cells)	N/A	N/A
Dengue Virus Type 1(in-house serotyped)	(Vaddadi et al.^50^)	

**Biological samples**		

Clinical serum samples (Dengue Infected patient serum)	Lotus Hospital for Women and Children, Lakdikapul-500004, Hyderabad, Telangana State, India.	N/A

**Chemicals, peptides, and recombinant proteins**		

DMEM	Gibco	Cat#11995-065
RPMI	Gibco	Cat#11875-093
FBS	Gibco	10270106
Anti-Anti (Antibiotic)	Gibco	Cat#15240062
Trypsin-EDTA (1X) solution	Himedia	Cat#TCL007
Paraformaldehyde	Sigma	Cat#P6148
Triton-X100	Himedia	Cat#TC286
RIPA Buffer	Sigma Aldrich	Cat# R0278
Hoechst stain 33342	Molecular probes by Life Technologies	Cat#H21492
Ni-NTA beads	Qiagen	Cat# 30210
NS2BNS3pro (recombinant protein)	(Gandikota et al.^47^)	N/A
Femto LUCENT™ PLUS-HRP	G-Biosciences	Cat. # 786-003
Lipofectamine reagent 2000	Invitrogen	Cat#11668-027

**Critical commercial assays**		

Midi prep plasmid isolation kit	Thermofischer Scientific	Cat#K0482lk
Gel Extraction Kit	Thermofischer Scientific	Cat# K0691

**Experimental models: Cell lines**		

Human Embryonic Kidney 293 cells (HEK 293)	National Centre for Cell Science [NCCS], Pune, India.	N/A
Vero cells (monkey kidney cell),	National Centre for Cell Science [NCCS], Pune, India.	N/A
Hepatocellular carcinoma (HepG2)	National Centre for Cell Science [NCCS], Pune, India.	N/A
Megakaryocyte derived cells (K562)	National Centre for Cell Science [NCCS], Pune, India.	N/A

**Oligonucleotides**		

NS2B FP- 5’TATGGGATCCGCTGATTTATC ATTGGAGAAA 3’	(Gandikota et al.^47^)	https://doi.org/10.1002/jmv.26024.
NS2B RP-5’CCCGCCTCCACCACTACCTCCGCC CCCGAGCGTGTCATCTCTCTCTTCAT 3’	Gandikota et al.^47^)	https://doi.org/10.1002/jmv.26024.
NS2B FP- 5’GCCCCTCAATGAAGGAATTATGG 3’	This paper	N/A
NS2B RP- 5’TGATCTCTGTTTCTTTTTCTGCCA 3’	This paper	N/A
NS3 FP-5’TATG*CTCGAG*ATGGGATGGTATCTATAGA 3’	This paper	N/A
NS3 RP- 5’ GGATGTAGGTCCATTATTGTTAGGT 3’	This paper	N/A
Helicase FP-5’ACATCCAGGATCAGGAAAAACA 3’	This paper	N/A
Helicase RP-5’TATC*GGATCC*CCCATGTAAATA TACTGG 3’ primers.	This paper	N/A
Overlapping Extension PCR NS2B FP 5’GCCCCTCAATGAAGGAATTATGG3’	This paper	N/A
Overlapping Extension PCR NS3 RP 5’ GGATGTAGGTCCATTATTGTTAGGT 3’	This paper	N/A
EDRF1 FP-5’TATC*GAATTC*GCCATGGCTGATT TGTCTACAGACTT 3’	This paper	N/A
EDRF1 RP-5’TATG*CTCGAG*CTGAAC GGCATTGCTGCT 3’	This paper	N/A

**Recombinant DNA**		

pRSET-NS2BNS3pro	Gandikota et al.^47^)	
pEGFP-N1 NS2BNS3pro	This paper	N/A
pEGFP-N1 NS2BNS3helicase	This paper	N/A
pEGFP-N1 NS3prohelicase	(Gandikota et al.^28^)	https://doi.org/10.1128/JVI.01178-20.
pCDNA 3.1 c-myc EDRF1	This paper	N/A
mitoRFP vector	(Gandikota et al.^28^)	https://doi.org/10.1128/JVI.01178-20.
pEGFP-N1 Vector	(Gandikota et al.^28^)	https://doi.org/10.1128/JVI.01178-20.

**Software and algorithms**		

Image J	ImageJ	https://imagej.nih.gov
Graph pad Prism version.9	Graph pad	https://www.graphpad.com
NIS ELEMENTS AR SOFTWARE	Nikon Microscopes	N/A
cNLS mapper software		https://nlsmapper.iab.keio.ac.jp

### Resource availability

#### Lead contact

Further information and reasonable requests for resources and reagents should be directed to the lead contact, Dr. Musturi Venkataramana (mvrsl@uohyd.ernet.in).

#### Materials availability

The recombinant constructs developed in the study will be available upon request.

#### Data and code availability

• All original data reported in this paper will be shared by the lead contact upon request.
• This paper does not report original code.
• Any additional information required to reanalyze the data reported in this paper is available from the lead contact upon request.

### Experimental model and study participant details

#### Cell lines

Human Embryonic Kidney 293 cells (HEK 293), Vero cells (monkey kidney cells), Hepatocellular carcinoma (HepG2) and Megakaryocyte derived cells (K562) were obtained from National Centre for Cell Science [NCCS], Pune, India. HEK, HepG2 and Vero cells were maintained in Dulbecco’s Modified Eagle’s medium (DMEM, Gibco, Invitrogen, USA). K562 cells were maintained in RPMI media (RPMI + L-Gluatamine, Gibco, Invitrogen, USA) at 37°C in a humidified incubator with 5% CO2. Both DMEM and RPMI were supplemented with 10% Fetal Bovine Serum (FBS) and 1% antibiotic containing (vol/vol) penicillin and streptomycin.

### Method details

#### Prediction of nuclear localization signal (NLS)

To identify the nuclear localization signal in NS2BNS3 and NS3, the amino acid sequences were retrieved from the NCBI data base of the in-house characterized DENV1 with Accession no. KX618706.1, Protein id. ASD49618.1. NLS in both the sequences were determined using the cNLS mapper software with a cutoff score of 0.4 and also identified the residues that show the monopartite and bipartite NLS sequences based on classical nuclear localization signal.^42^

#### Dengue virus constructs

All the constructs were generated from the in-house characterized DENV1 serotype . pRSET-A NS2BNS3pro (46a.a+185a.a.) clone was generated earlier in our previous study.^47^ NS2BNS3pro consists of hydrophilic domain of NS2B (46a.a) and protease domain of (NS3 185a.a) were linked via a G4-S-G4 linker sequence. NS2B was amplified using NS2B Forward- 5’ TATG*GGATCC*GCTGATTTATCATTGGAGAAA and NS2B Reverse – 5 CCCGCCTCCACCACTACCTCCGCCCCCGAGCGTGTCATCTCTCTCTTCAT 3’ primers. NS3pro was amplified using NS3 Forward-5’GGGGGCGGAGGTAGTGGTGGAGGCGGGAGAGCAGTTCTTGATGATGGTA and NS3 Reverse 5’ATCGA*GAATTC*TTACCTAAACACCTCGTCCTCAATC3’ primers. The obtained gene products were used as templates with external primers (the above forward primer of NS2B and the reverse primer of NS3) for overlap extension PCR to generate NS2B-G4-S-G4-NS3pro. This amplified DNA was digested with *ECoRI* and *BamHI* enzymes and then ligated to the pRSET-A vector. The clone was confirmed by sequencing. In order to develop pEGFP-N1 NS2BNS3pro, the above pRSET-A NS2BNS3pro was used as a template with forward 5’ TATG*CTCGAG*ATGGCTGATTTATCATTGGAC 3’ and reverse 5’ TATC*GGATCC*GTAAACACCTCGTCCTC 3’ primers. The amplified fragment (696 bp) and pEGFP-N1 vector were digested with *XhoI/BamHI* enzymes and gel purified by gel extraction kit (Thermofisher Scientific). The digested products were ligated using T4 ligase enzyme. Ligated product was transformed into *E. Coli*. DH5*α* competent cells and the isolated plasmid was confirmed with restriction digestion using *XhoI/BamHI* enzymes. The positive clone pEGFP-N1 NS2BNS3pro was further confirmed by sequencing*.* pEGFP-N1 NS3pro-helicase (464a.a) construct was also developed during our previous study.^28^ To develop this construct, NS3pro (185a.a) was amplified using the NS3 forward 5’ TATG*CTCGAG*ATGGGATGGTATCTATAGA3’ and reverse 5’ GGATGTAGGTCCATTATTGTTAGGT 3’ primers. Helicase was amplified using forward F 5’ACATCCAGGATCAGGAAAAACA 3’ and reverse 5’TATC*GGATCC*CCCATGTAAATATACTGG 3’ primers. The reaction was performed in a final volume of 25μl containing 0.5 μl cDNA template, 2.5 μl reaction buffer, 0.5μl of 10mM dNTPs, 0.25U of Taq polymerase enzyme, 0.5 μl of each of 10 μM forward and reverse primers. Cycling conditions were followed with an initial denaturation step at 95°C for 4 minutes, followed by 35 cycles of 94°C for 30 seconds, 53°C for 30 seconds and 72°C for 1 minute, and final extension at 72°C for 10 minutes. The final products were electrophoresed on 1% agarose gel and purified by gel extraction method. Further overlapping extension PCR was carried to generate NS3pro-helicase (464a.a) using forward primer of NS3 protease containing *XhoI* restriction site at 5’ end and reverse primer of helicase containing *BamHI* restriction site at 3’ end as mentioned above. The plasmid construct, NS3pro-helicase consists of 185 amino acids protease domain of NS3 and 283 amino acids of helicase domain. The obtained clone was ligated into pJET 1.2 vector and further subcloned into pEGFP-N1 vector using *XhoI/BamHI* restriction sites to obtain pEGFP-N1 NS3pro-helicase (464a.a).

In order to develop pEGFP-N1 NS2BNS3 (130+464=594 a.a), NS2B fragment was amplified using NS2B forward 5’ GCCCCTCAATGAAGGAATTATGG 3’ and NS2B reverse 5’ TGATCTCTGTTTCTTTTTCTGCCA 3’ primers. The reaction was performed in a final volume of 25μl containing 0.5μl of cDNA template, 2.5μl reaction buffer, 0.5μl of 10mM dNTP mix, 0.25U of Taq polymerase enzyme, 0.5μl of each 10 μM forward and reverse primers. The pTZ57RT-NS2BTA construct that existed in lab was used as a template. Cycling conditions were followed with an initial denaturation step at 95°C for 4 minutes, followed by 35 cycles of 94°C for 30 seconds, 53°C for 30 seconds, 72°C for 1 minute, and final extension at 72°C for 10 minutes. The final products were electrophoresed on 1% agarose gel and purified by gel extraction method and used as templates for overlapping extension PCR. The reaction mixture was prepared by adding the templates, NS2B and NS3pro-helicase (100ng each), 1X buffer, 10mM dNTP and Q5 polymerase. Two step overlapping extension PCR was carried out to join NS2B at the 5’ end of NS3pro-helicase: 94°C for 4 minutes, (94°C for 30 seconds, 55°C for 40 seconds, 72°C for 1.4 minutes) 10 cycles followed by 72°C for 15 minutes as a final extension. Later, external primers (NS2B forward & helicase reverse) were added and followed by 35 cycles of second round of PCR, 94°C for 30 seconds, 53°C for 30 seconds, 72°C for 1.4 minutes, and final extension at 72°C for 10 minutes. The obtained fusion fragment was electrophoresed on 0.8% agarose gel and purified by gel extraction method. The overlapping extension PCR amplified product of NS2BNS3 (594 a.a) was ligated into pJET 1.2 using T4 DNA ligase. The ligated product was transformed into *E. coli*. DH5*α* competent cells. Plasmid was isolated, and insert was confirmed by restriction digestion. To sub-clone pJET 1.2 NS2BNS3 into pEGFP-N1 vector, pJET 1.2 NS2BNS3 and pEGFP-N1 vector were subjected to *XhoI/HindIII* restriction enzyme digestion. 1.8 kbp digested product from pJET1.2 clone was obtained and further ligated to linearized pEGFP-N1 vector. The ligated recombinant was transformed into *E. coli*. DH5*α* competent cells. Plasmid was isolated, and insert was confirmed by restriction digestion and further by sequencing. To clone NS2BNS3pro (S135A) mutant (130+185a.a), NS3pro (S135A) was generated by site directed mutagenesis. The dengue virus NS3 protease inactive mutant was generated by amino acid substitution Ser135 in catalytic triad with Ala using the primers Forward, 5’-TTTTAAACCCGGCACAGCTGGATCTCCC 3’ and Reverse, 5’-TCACGATGGGAGATCCAGCTGTGCCGG 3’. Above amplified NS2B (130a.a) and NS3pro (S135A) (185a.a) were overlapped with overlapping extension PCR using forward 5’ GCCCCTCAATGAAGGAATTATGG 3’ of NS2B at 5’ end and reverse 5’ GGATGTAGGTCCATTATTGTTAGGT 3’ primers of NS3 at 3’ end as described above. The obtained fusion fragment was electrophoresed on 0.8% agarose gel and purified by gel extraction method. NS2BNS3pro (S135A) mutant was ligated to pJET 1.2 blunt end vector using T4 ligase and clone was confirmed using *XhoI/HindIII* restriction digestion. The digested NS2BNS3pro (S135A) mutant was further subcloned to pEGFP-N1 vector as described above. NS2BNS3 (594 a.a) and NS3pro-helicase (464a.a) are the naturally existing forms of dengue virus infections.^12^

#### Cloning of EDRF1 in pcDNA3.1 c-myc vector

From K562 cell pellet, total RNA was isolated using Trizol as per manufacturer’s protocol. For cDNA synthesis, total isolated RNA was mixed with oligo(dT) primers in sterile RNase free water and reaction was followed up with mixing: total RNA (1μg), oligo(dT) random primer mix (0.5μg/μl), 10 mM dNTP, and RNase free water. The RNA was denatured for 5 minutes at 65°C. Then 10X AMV buffer, AMV Reverse Transcriptase (10U/μl), RNase inhibitor (40U) and RNase free water were added. Total RNA was reverse transcribed using the reaction cycle: 45°C for 50 minutes, 80°C for 5 minutes. The cDNA generated was used as template for amplification of EDRF1 with forward 5’ TATC*GAATTC*GCCATGGCTGATTTGTCTACAGACTT 3’ and reverse 5’ TATG*CTCGAG*CTGAACGGCATTGCTGCT 3’ primers. The primers were designed based on the EDRF1 sequence available with accession no NP_001189367.1. The PCR mix contains 10X buffer, 10mM dNTP, forward and reverse primers (10μM each), cDNA (100ng), Taq Polymerase (0.25U) and water. Cycling conditions were followed with an initial denaturation step at 94°C for 5 minutes, followed by 35 cycles of 94°C for 1 minute, 58°C for 30 seconds, 72°C for 1.5 minutes, and final extension at 72°C for 10 minutes. The amplified fragment was run on 1% agarose gel and 1.3 kbp amplified product was obtained. To further clone the amplified fragment in pcDNA3.1 c-myc vector, both the vector and insert were digested with restriction sites *XhoI*/*ECoRI*, ligated and transformed into *E. Coli*. DH5*α* competent cells. The recombinant plasmid was isolated and further confirmed with restriction digestion and sequencing.

#### Dengue virus protease localization analysis

Transient transfections were performed using the constructs pEGFP-N1 vector, pEGFP-N1 NS2BNS3pro (46a.a+185a.a), pEGFP-N1 NS2BNS3pro (S135A) mutant (130 a.a + 185 a.a) and pEGFP-N1 NS2BNS3 (130 a.a + 464 a.a) in K562 cells. Briefly, one day before transfection, approximately 10^5^ cells were seeded in 6-well plate. Transfection was performed with the above mentioned constructs using Lipofectamine 2000 reagent as per manufacture’s protocol in ratio (1.5μg plasmid and 3μl Lipofectamine). After 28 hours the cells were harvested, washed with 1X PBS and fixed using 4% paraformaldehyde for 20 minutes. Then the cells were permeablized with 0.25% Triton-X 100 in 1X PBS for 5 minutes, washed with 1X PBS followed by nuclear staining with Hoechst 33342 stain (Molecular probes). The cells washed and resuspended in 1X PBS and Glycerol (1:1) and mounted on to glass slide with a coverslip. The cells were analyzed using laser scanning confocal microscope (Carl Zeiss) at 20X and 100X magnifications. The images were merged in order to observe the co-localizations. The localization color (green/blue) intensity profiles were prepared using NIS Elements AR software program to analyze the GFP (green peak) in nucleus along with Hoechst stain (blue). The fluorescence percentage was calculated using (Mean intensity of each channel/total intensity in nucleus X100). *p*- values indicated p<0.0001, ∗∗∗∗ = significant, ns = non-significant.

In order to analyze the cross localizations of NS2BNS3 (in mitochondria) and NS3pro-helicase (in nucleus), we have used MitoRFP vector and Hoechst satin for both the constructs. In this direction, transient transfections were performed in HEK cells using the constructs pEGFP-N1 vector, pEGFP-N1 NS2BNS3 (594 a.a) and pEGFP-N1 NS3pro-helicase (464 a.a) along with MitoRFP vector. HEK cells were seeded onto coverslips in 12 well plate and the plasmid constructs were transfected along with mitoRFP vector (1μg to 2μl Lipofectamine). After 48 hours, the cells were washed with 1X PBS and fixed using 4% paraformaldehyde for 20 minutes and permeablized with 0.25% Triton-X 100 in 1X PBS for 5 minutes, followed up with staining with Hoechst stain (nucleus) and mounting the coverslip with glycerol on to glass slide. The cells were analyzed using laser scanning confocal microscope at 60X magnification and the images were merged for the analysis. The localization intensity profiles were prepared using NIS Elements AR software program. The fluorescence percentage was calculated as above.

#### Detection of dengue virus protease in subcellular fractions

Similar transfections were performed (pEGFP-N1 vector, pEGFP-N1 NS2BNS3 (594 a.a) and pEGFP-N1 NS3pro-helicase (464 a.a)) as described above in HEK cells. For preparing whole cell lysates, cells were harvested and washed with 1XPBS by centrifuging at 3000rpm for 5 minutes. The cell pellet was resuspended in RIPA buffer, incubated on ice and vortexed 2-3 times in intervals for 15 minutes. The solubilized lysate was centrifuged at 14000rpm for 15 minutes and the supernatant was collected as whole cell lysate. For preparing cytoplasmic and nuclear fractions, cells were washed with ice-cold 1X PBS, mildly homogenized in hypotonic buffer (20mM HEPES, 10mM KCl, 1mM EDTA, 10% glycerol and 0.5% Triton-X100) and incubated for 10 minutes. The homogenized lysate was centrifuged at 2000g at 4°C for 10 minutes. The supernatant collected as cytoplasmic fraction and the pellet was used for nuclear extraction. The pellet was further washed twice with 1XPBS and resuspended in RIPA buffer. The resuspended pellet was incubated for 30 minutes on ice and vortexed at 5 minute intervals. The completely dissolved pellet was allowed for centrifugation at 20,000g for 15 minutes. The supernatant was collected as a nuclear fraction. The lysates were quantified using Bradford reagent and resolved on 10%SDS PAGE and immunoblotted with NS2BNS3pro antibody (1:1000), anti-GFP antibody (1:1000) and actin (1:2500).

#### *In vitro* pulldown assay and protein identification

K562 cells were cultured in 100mm dishes. The cells were harvested and lysed with RIPA buffer. The lysate was quantified by Bradford reagent (Bio-Rad) and 250-300μg protein (500μl) lysate was used for pulldown. pRSET-A NS2BNS3pro purified protein (200μg) was allowed for binding to Ni-NTA His beads for 3 hours with gentle rocking and centrifuged for 1 minute at 4°C to remove the unbound protein. K562 cell lysate incubated with the protein bead complex overnight with gentle rocking at 4°C. The complex was centrifuged for 1 minute and unbound proteins were collected as flow through (FT), followed by washes of 10-50mM imidazole (500μl each). The interacting proteins were eluted from washed bead complex using imidazole (100-300mM).

#### Identification of proteins obtained in the above pull-down experiment

Elutes, FT washes and the bead complex were mixed with 4X Laemmli buffer and boiled for 10 minutes, centrifuged for 1 minute and resolved on 10% SDS PAGE. A similar procedure was followed up with Ni-NTA beads bound to K562 cell extract without purified protein (control). The gel containing the bands were eluted and followed up by MALDI TOFF mass spectrometry analysis (Galaxy International /Sandor Life Sciences Pvt. Ltd.). Further, the above-mentioned flow through, washes and elutes were resolved on 10% SDS PAGE, transferred onto PVDF membrane and probed with anti-EDRF1 antibody (1:3000). The anti-rabbit secondary HRP conjugated antibody was used (1:10000) and the developed image was recorded (Chemidoc, Bio-Rad).

#### Identification of protease cleavage sites in EDRF1 using *in silico* methods

Full length EDRF1 protein sequence accession no: Q3B7T1.1, was retrieved from NCBI data base. Protease cleavage sites were identified by using ProP-1.0 Server-DTU Health Tech. Further, the protease and EDRF1 structures were superimposed using auto docking tool to identify the cleavage sites for the protease catalytic triad.

#### EDRF1 cleavage analysis with co-transfection studies

HEK cells were seeded and allowed to grow up to 80% confluency. pEGFP-N1 NS2BNS3pro (46 a.a + 185 a.a) was transiently co-transfected with pcDNA3.1 c-myc EDRF1. Transfection was done using Lipofectamine 2000 reagent (1:3 ratios) as per the manufacturer’s protocol. After 48 hours, cells were harvested and proceeded for western blotting and the cleavage was analyzed using anti-Myc tag (1:1000) and anti-GFP antibodies (1:1000). Secondary HRP-conjugated anti-mouse and anti-rabbit antibodies (1:10000), respectively were used.

#### Analysis of EDRF1 levels in transfected cell extracts

Cells were seeded at conc. of 5x10^5^ cells/ml in 6 well plates prior to the day of transfection in RPMI growth media with 10% FBS without antibiotic and incubated at 37°C with 5% CO_2_. After 12 hours, cells were transfected with pEGFP-N1 vector, pEGFP-N1 NS2BNS3pro, pEGFP-N1 NS2BNS3pro (S135A) mutant, pEGFP-N1 NS2BNS3 (1-5μg/μl) and allowed to grow for 24 hours. Cells were analyzed for GFP expression using fluorescence microscope. Then the cells were harvested and cell extracts prepared, resolved on 10% SDS PAGE and probed with anti-GFP (1:1000) or anti-NS2BNS3pro (1:5000) or anti-EDRF1 (1:3000) or anti-GATA1 (1:5000) or anti-alpha1 spectrin (1:2500) antibodies (one blot with anti-GFP or anti-NS2BNS3pro or anti-EDRF1; the other blot with anti-GATA1 or anti-alpha1 spectrin). The secondary anti-rabbit HRP-conjugated antibody (1:10000) was used and anti-actin (1:2500) was used and probed with HRP-conjugated anti-mouse secondary antibody (1:10000). The blot was developed and imaged using Chemidoc (Bio-Rad).

#### Analysis of EDRF1 levels in virus infected cell extracts

Virus infection in Vero cells was performed as described in our earlier studies.^28^ For infection of K562 cells, Vero cells propagated viral supernatant was used. A day before infection four dishes of size 100mm were seeded with 10^6^ cells. On the day of infection, cells were harvested and counted using a hemocytometer. Suspension cells were seeded again in 60mm (10^5^) cells per dish and inoculated with viral supernatant in ratio 1:4 dilutions with MoI of 0.09 in serum free RPMI media. The virus was allowed for adsorption for 2-3 hours with mild agitation in 15 minutes’ intervals. After the adsorption, the media was replaced with RPMI media containing 5% FBS and cells were allowed to grow for up to 7 days. Uninfected K562 cells were used as control which were also allowed to grow for 7 days. Infection was confirmed with NS2BNS3pro antibody (1:1000). The cells were harvested, cell lysate was prepared and western blotting was carried using anti-EDRF1, anti-GATA1 and anti-alpha1 spectrin antibodies as described above.

#### EDRF1 and GrpEL1 levels in clinical samples

Clinical samples (n=44) were classified as DF, DHF and DSS as per WHO guidelines (Table S1). The samples were processed for albumin depletion. Briefly, in 100μl of serum sample, 1M NaCl was added to final conc of 0.1M and incubated for 60 min at 4°C on rocking. Ice cold ethanol was added to the sample to a 42% concentration and further incubated for 60 min at 4°C. The sample was centrifuged at 14,000 rpm for 45 min at 4°C, and the pellet was stored. Using 0.8 M cold sodium acetate (pH 4.0), the pH of the supernatant was lowered to 5.7 and incubated for 30 min at 4°C. Again, the sample was centrifuged at 14,000 rpm for 30 min, and the supernatant containing albumin was separated. Both pellets were re-suspended and mixed in 10 mM Tris, pH 6.8 and 1 M urea. The protein concentrations of obtained albumin-depleted samples were estimated by Bradford assay. Samples (10μg) were resolved on a 10% SDS-PAGE gel and immunoblotted with anti-EDRF1 antibody (1:3000) followed by stripping and probed with anti-GrpEL1 antibodies (1:2500). Anti-rabbit secondary HRP-conjugated antibodies (1:10000) were used and the blot was developed as described above.

#### Bioenergetics of mitochondria in transfected cells

Transient transfection was performed in HepG2 cells with plasmid constructs pEGFP-N1 vector and pEGFP-N1 NS3pro-helicase as per manufacturer’s protocol. 28 hours post transfection 7000-8000/well cells were seeded in Agilent Seahorse XFp mini culture plates (8-wells) up to 70% confluency and allowed to grow at 37°C in 5% CO2 incubator, as per the manufacturer’s protocol. For the cell mitostress assay, the sensory cartridge was hydrated with milli-Q water one day before at 37°C incubator without CO_2_. Next day, sensory cartridge is equilibrated with XFp Calibrant solution for 2 hours. Later, the sensory cartridge was filled with three drugs [Oligomycin (1.5μM), FCCP (0.5 μM), Rotenone/Antimycin (0.5 μM)] as per protocol and XFp cell mitostress assay was performed using Seahorse XFp Analyzer with the in-built software program.^48^^,^^49^

#### Cell number analysis during dengue virus infection

2 sets of 60mm dish (10^5^) K562 cells (control, infected) were infected with viral supernatant for 0, 3, 5 and 7 days, harvested and counted manually with trypan blue dye (Sigma) using a hemocytometer. The virus infection was confirmed by RT-PCR of 5’ UTR on 5^th^ day. The experiments were performed for three times, the total cell count after each day of infection was taken and an average of two experiments were considered using Microsoft Excel software.

### Quantification and statistical analysis

The graphs were presented using graph pad prism 9. Experiments were performed at least 3 times and data were represented as means and standard deviations and were statistically analyzed by Student’s *t-tests* and one-way ANOVA analysis. (∗), (∗∗), (∗∗∗) signifies p-values of 0.05,0.005 and 0.0001. (ns) signifies non-significant. All densitometry analysis was done using Image J software.

## Supplemental information

Document S1. Figures S1–S4 and Table S1

Video S1. The audio-embedded video explains the NS2BNS3 & NS3 subcellular localizations and consequences leading to thrombocytopenia, related figures 2 (A&B), 3 (A&B), 4 (H), 5, 6, and 7
