# Supplementary material for: Differential localization of dengue virus protease affects cell homeostasis and triggers to thrombocytopenia
Source: iScience. 2023 Jun 5;26(7):107024. doi: 10.1016/j.isci.2023.107024 (PMC10391676; doi:10.1016/j.isci.2023.107024)
Supplement: Document S1. Figures S1–S4 and Table S1 [file mmc1.pdf]

iScience, Volume *26*

Supplemental information

Differential localization of dengue virus protease affects cell homeostasis and triggers to thrombocytopenia

Lekha Gandhi, Deepti Maisnam, Deepika Rathore, Preeti Chauhan, Anvesh Bonagiri, and Musturi Venkataramana

Figure S1. [Representative images of transfected cells (analysed using the confocal microscope at 20X-100µm) showing the localization of expressed proteins]**, Related to Figure 2 and STAR methods.**

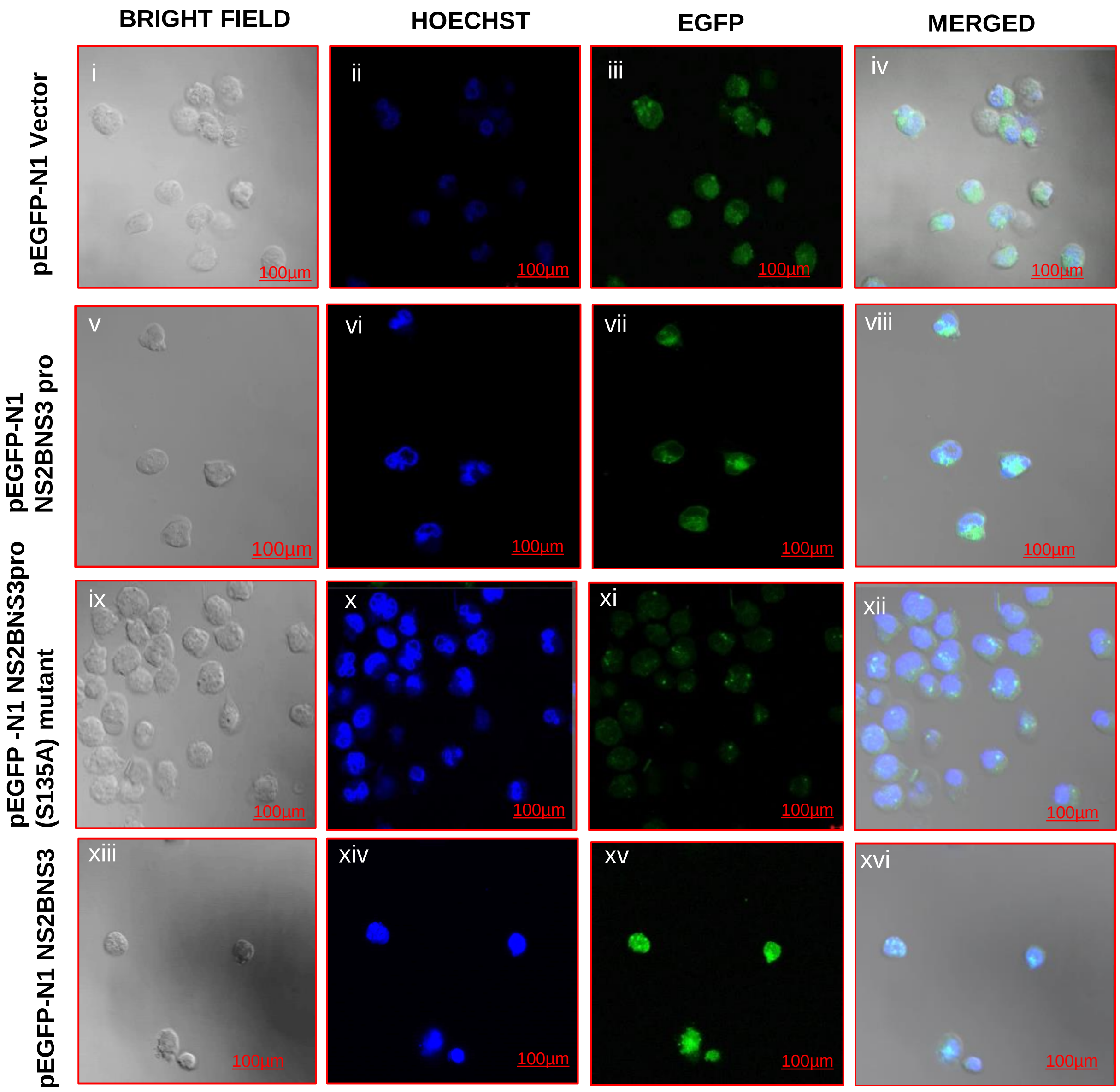

K562 cells transfected with pEGFP- N1 vector (i-iv), pEGFP N1 NS2BNS3pro (v-viii); pEGFP N1 NS2BNSpro (S135A) mutant (ix-xii); pEGFP N1 NS2BNS3 (xiii-xvi).

Figure S2. [(A&B) 10% SDS-PAGE analysis of proteins obtained by *In vitro* pull down experiment], **Related to Figure 4A.**

A)

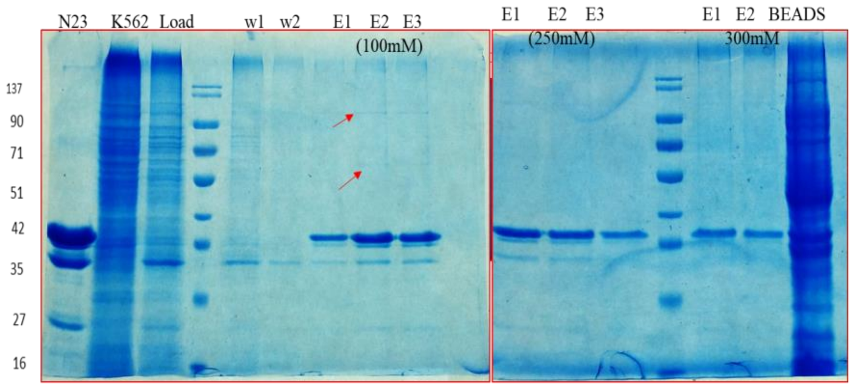

B)

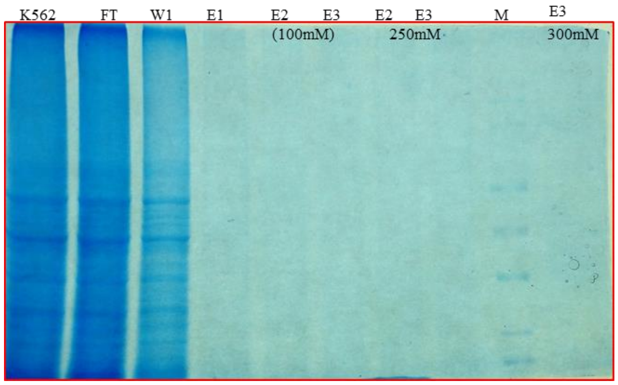

A.) Cell lysate with NS2BNS3pro purified protein (protein bands indicated with arrows in E2&E3 are identified. **B)** Negative control without the NS2BNS3pro protein.

C) [Represents the amino acid sequence of EDRF1 showing the protease cleavage sites (red)], **Related to Figure 4 H-J and STAR methods**

EDRF1 : (Q3BT71)

>sp|Q3B7T1.1| EDRF1_HUMAN Full=Erythroid differentiation-related factor 1

MGDAKEAGAEGPPAGAAARGGLSLLSQGESEESSAQGSALFLGGNEVKSRAVVKYSSAPPRTAFARLEEKTDLKLPP
ANWLRESAKLGPAGTTILGNSKKSKPFSSFGMAYDFIDSVGNDVDVVSDSENIKKLLKIPYSKSHVSMAVHRIGRTLLL
DELDIQELFMRSSQTGDWTWLKEFYQRLIDQKWQRKKKSKEHWYQKAILSKFLYYSINGDGAAQPVSSTAEQQESSS
SDQTNDSEGASWPAPFEMPSSVSEDPSASSQGSEPLEPSYIVGHVASAPKEQNLITLFNDGEHSQGLKNDFVRNILW
TFEDIHMLVGSNMPIFGGGRYPAVSLRLRDNNKPINVLTGIDYWLDNLICNVPELVMCFHVNGIVQKYEMIKTEEIPNLE
NSNFSTKVIKDIAQNILSFLKSNCTKEGHTYWLFKASGSDIVKLYDLTTLCEETEDKYQNPFTMPVAILLYKVACNMMMK
KNQNKKHYGTIRTLLLNCLKLLDKSRHPQIIASANYMLSELFQLDEPKKEENSESPLNENSDESYSEEEEEMPDSDENG
SYSTSSDPSDDSKAVAIIKSVGELSVPEKYKSIHQIRPSCAFPVCHDTEERCRLVLSYVLEGLKSVDSSIKKESDLPAAD
PSTPIPLKYEDESSRGGPEGLEKQMALFLDKMGSLQKGNYSSQSGMIPGSWQHKMKLQLILKSSKAYYVLSDAAMSL
QKYGRALRYIKLALQSHDTYCCLCTNMLSEVLLFLSQYLTLCGDIQLMLAQNANNRAAHLEEFHYQTKEDQEILHSLHR
ESSCQGFAWATDLSTDLESQLSVSCKCYEAANEILQFSDLKSQNPEHYVQVLKRMGNIRNEIGVFYMNQAAALQSER
LVSKSVSAAEQQLWKKSFSCFEKGIHNFESIEDATNAALLLCNTGRLMRICAQAHCGAGDELKREFSPEEGLYYNKAID
YYLKALRSLGTRDIHPAVWDSVNWELSTTYFTMATLQQDYAPLSRKAQEQIEKEVSEAMMKSLKYCDVDSVSARQPL
CQYRAATIHHRLASMYHSCLRNQVGDEHLRKQHRVLADLHYSKAAKLFQLLKDAPCELLRVQLERVAFAEFQMTSQN
SNVGKLKTLSGALDIMVRTEHAFQLIQKELIEEFGQPKSGDAAAAADASPSLNREEVMKLLSIFESRLSFLLLQSIKLLSS
TKKKTSNNIEDDTILKTNKHIYSQLLRATANKTATLLERINVIVHLLGQLAAGSAASSNAVQ

*Cleavge sites identified by the ProP1 server.

Figure S3. [(A&B) *In silico* superimposed models of EDRF1 (Cyan) and NS3pro-helicase (green)], **Related to Figure 4 H-J and STAR methods.**

A)

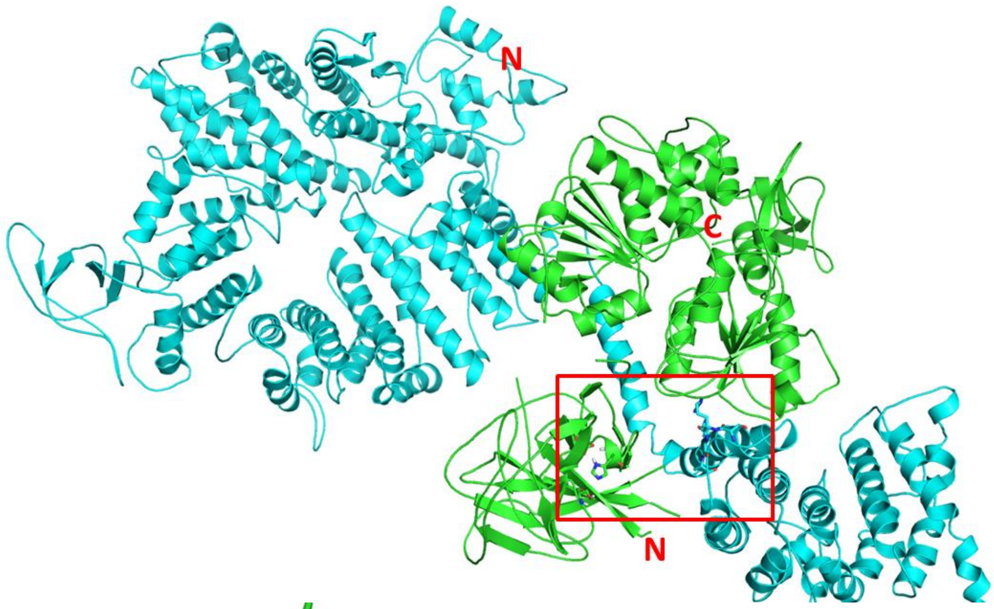

B)

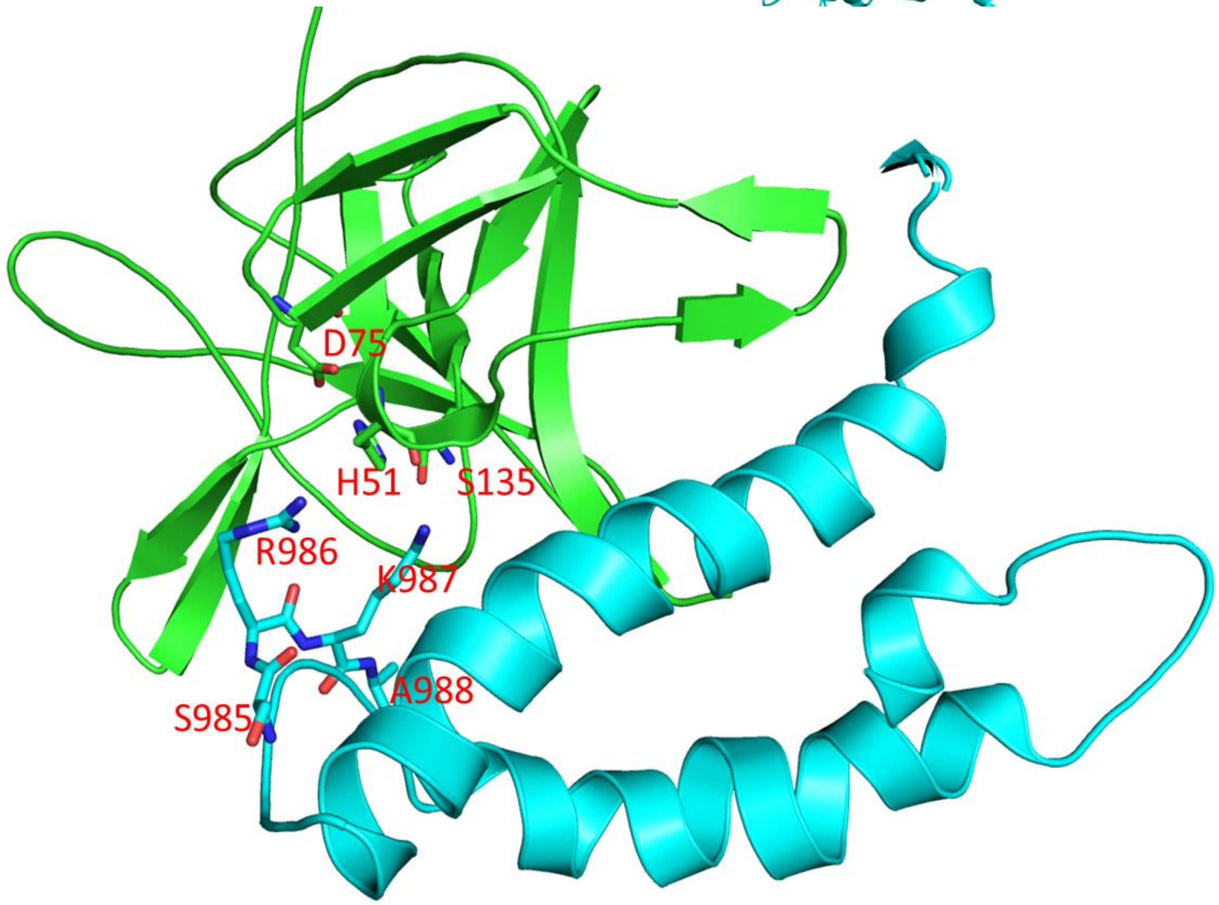

A) The *in silico* superimposed models of EDRF1 (Cyan) and NS3pro-helicase (green). B) Enlarged version of the superimposed area showing the interactions of catalytic triad of NS3pro-helicase (H-51, D-75, S-135) and the cleavage site at a.a 985-988 (RK987A).

Figure S4. [(A&B) Western blot analysis of clinical samples using anti-EDRF1 antibodies], **Related to Figure 5L, and STAR methods.**

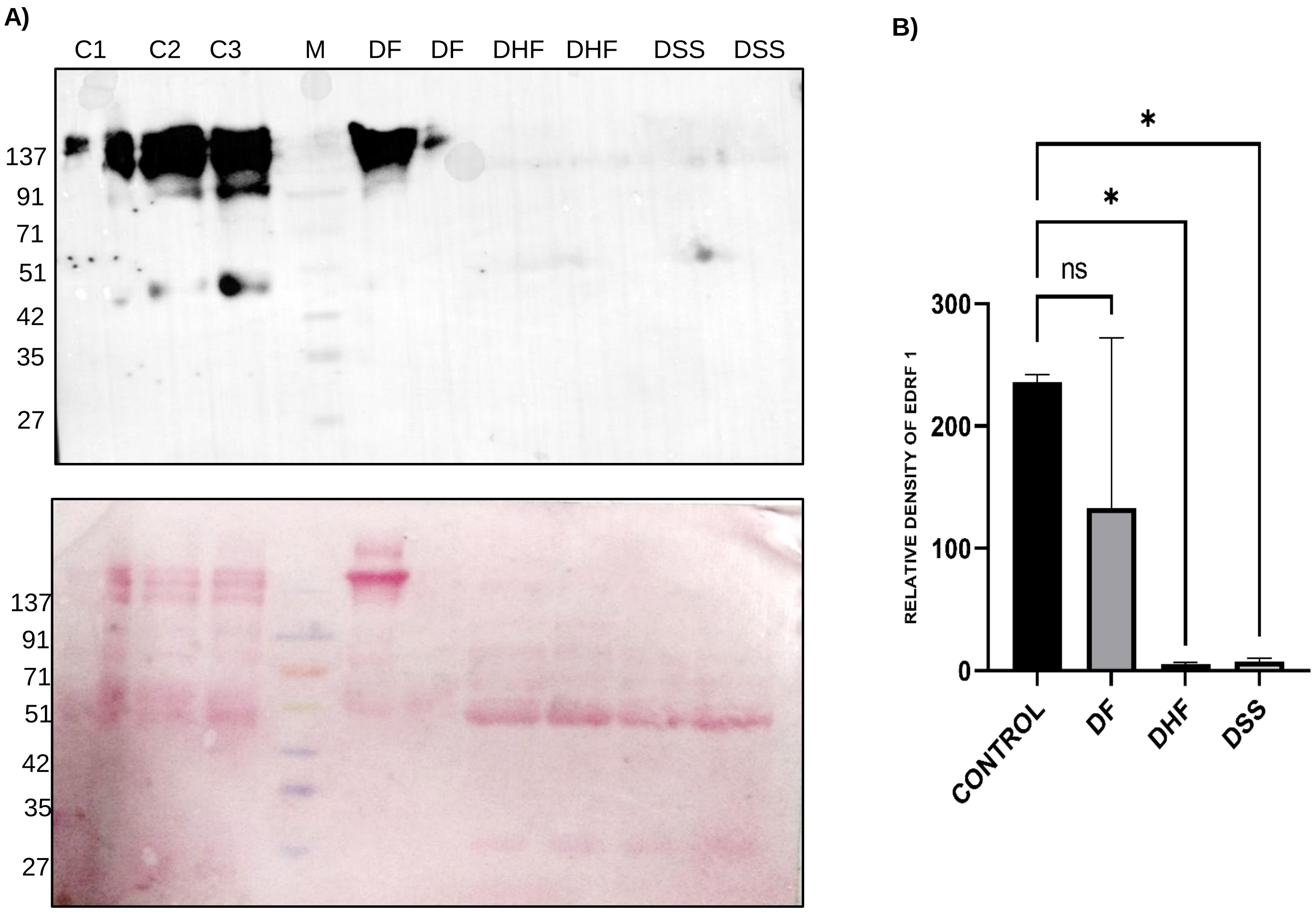

(A) Albumin out other febrile (control) and dengue infected serum samples (DF, DHF and DSS) were separated on SDS PAGE and western blotting was done to analyze the EDRF1 levels. Bottom panel represents the Ponceau S stained membrane. (B) Bar diagram represents the relative density of EDRF1 protein in clinical samples. (*) signifies p-values < 0.05 and ns- non-significant.

Table S1: [Clinical characteristics of dengue virus infected patient samples used in the study], **Related to Figure 5L and STAR Methods. A)** Clinical features, **B-D)** Samples classified as DF, DHF, DSS.

**A.) Clinical features**	**B.) Dengue fever (n=24)**	**C.) Dengue Hemorrhagic Fever (n=17)**	**D.) Dengue Shock Syndrome (n=4)**
GENDER (MALE/FEMALE)	11/13	9/8	3/1
AGE	<1Year to 14 years	<1 year to 15years	<1 year to 12 years
NS1 POSITIVE	15	15	4
IgM/IgG	10	8	1
Thrombocytopenia	19	14	4
Platelet count	(>6000 to <1,50,000)*	(>2000 to <2,30,000)*	(>5000 to <1,50,000)*

Platelet count were analysed based on the clinical data sheets. * Represents the range of platelet count.
